# CYRI-A limits invasive migration through macropinosome formation and integrin uptake regulation

**DOI:** 10.1083/jcb.202012114

**Published:** 2021-06-24

**Authors:** Anh Hoang Le, Tamas Yelland, Nikki R. Paul, Loic Fort, Savvas Nikolaou, Shehab Ismail, Laura M. Machesky

**Affiliations:** 1 Cancer Research UK Beatson Institute, Bearsden, Glasgow, UK; 2 Institute of Cancer Sciences, University of Glasgow, Bearsden, Glasgow, UK; 3 Department of Cell and Developmental Biology, Medical Research Building III, Vanderbilt University, Nashville, TN; S. Ismail’s present address is Department of Chemistry, Katholieke Universiteit Leuven, Heverlee, Belgium.

## Abstract

The Scar/WAVE complex drives actin nucleation during cell migration. Interestingly, the same complex is important in forming membrane ruffles during macropinocytosis, a process mediating nutrient uptake and membrane receptor trafficking. Mammalian CYRI-B is a recently described negative regulator of the Scar/WAVE complex by RAC1 sequestration, but its other paralogue, CYRI-A, has not been characterized. Here, we implicate CYRI-A as a key regulator of macropinosome formation and integrin internalization. We find that CYRI-A is transiently recruited to nascent macropinosomes, dependent on PI3K and RAC1 activity. CYRI-A recruitment precedes RAB5A recruitment but follows sharply after RAC1 and actin signaling, consistent with it being a local inhibitor of actin polymerization. Depletion of both CYRI-A and -B results in enhanced surface expression of the α5β1 integrin via reduced internalization. CYRI depletion enhanced migration, invasion, and anchorage-independent growth in 3D. Thus, CYRI-A is a dynamic regulator of macropinocytosis, functioning together with CYRI-B to regulate integrin trafficking.

## Teaser

Le et al. implicate CYRI-A in resolving macropinosome formation by locally sequestering active RAC1. They further show that CYRI-mediated macropinocytosis contributes to integrin internalization, impacting spreading and invasion of cancer cells.

## Graphical Abstract

**Figure:**
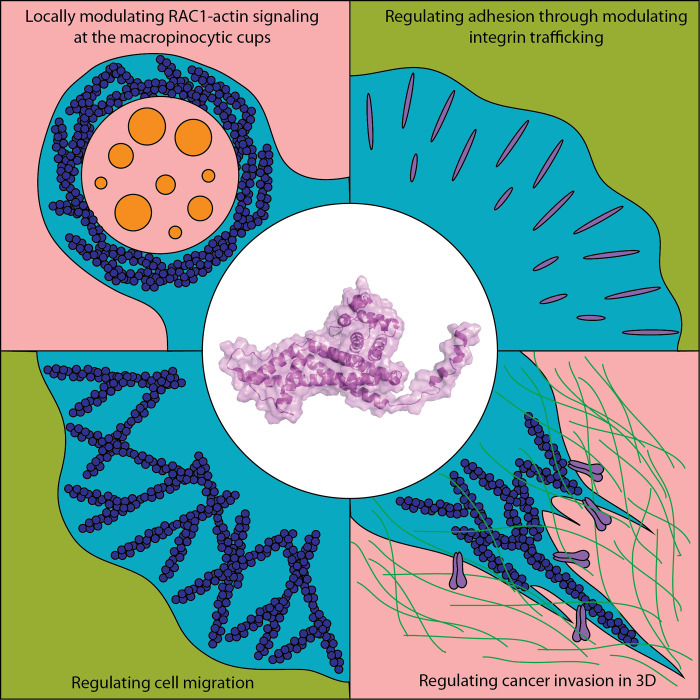

## Introduction

The actin cytoskeleton is a multifaceted network coordinating essential cellular processes such as cell migration and endocytosis. The small GTPase RAC1 acts as a regulatory switch at the heart of this network. By activating the Scar/Wiskott Aldrich Syndrome Family verprolin homologous protein (WAVE) complex, RAC1 indirectly triggers Arp2/3 complex activation and thus branched actin polymerization.

RAC1-induced actin polymerization contributes to endocytic processes (Mooren et al., 2012; Egami et al., 2014; Bloomfield and Kay, 2016; Ferreira and Boucrot, 2018; Hinze and Boucrot, 2018). The branched actin network underpins membrane ruffling, a prerequisite for macropinocytosis, an ancient endocytic pathway for uptake of external substances, or to counteract cytoplasmic hydrostatic pressure to drive membrane curvature and invagination (Carlsson, 2018). Evidence suggests that a balanced spatial and temporal regulation of RAC1 and its downstream cytoskeletal targets at the site of endocytosis is essential. Following RAC1 activation, RAC1 inactivation is required for the completion of macropinocytosis (Yoshida et al., 2009; Fujii et al., 2013). Constitutively activating RAC1 by photoactivation led to unresolved membrane invagination (Fujii et al., 2013). The related process phagocytosis has been shown to require several GTPase-activating proteins (GAPs) in the deactivation phase of RAC1 but only for particles >8 µm (Schlam et al., 2015). However, the mechanism of RAC1 deactivation in macropinocytosis remains largely unknown.

Integrins are type I transmembrane proteins important in cell adhesion and migration, and their endocytic trafficking is implicated in cancer cell invasion and metastasis (Caswell et al., 2009; Cooper and Giancotti, 2019). In breast cancer, increased surface expression of integrin α5 increases the cell contraction force and invasion (Mierke et al., 2011). In ovarian cancer cells, integrin α5β1 is transported toward the invasive front by the Wiskott Aldrich Syndrome Family and Scar homologue complex to increase invasion in 3D (Zech et al., 2011). Among the trafficking routes, such as clathrin- or caveolin-1–dependent endocytosis (Shi and Sottile, 2008), bulk internalization pathways such as macropinocytosis and the CLIC-GEEC (Clathrin-independent carriers–glycophosphatidylinositol enriched early endosomal compartment) pathway are involved in integrin trafficking and can affect the migration and invasion behavior of cells (Gu et al., 2011; Moreno-Layseca et al., 2020 *Preprint*).

Recently, we identified CYFIP-related RAC1 interacting (CYRI) proteins as negative regulators of the Scar/WAVE complex by RAC1 sequestration (Fort et al., 2018). Actin polymerization by the Scar/WAVE complex also drives the uptake of cellular pathogens and nutrients by macropinocytosis and phagocytosis (Veltman et al., 2016; Humphreys et al., 2016). Indeed, CYRI-B protects against *Salmonella* infection (Yuki et al., 2019), regulates T cell activation (Shang et al., 2018), and potentially acts as a tumor suppressor (Chattaragada et al., 2018). Mammalian cells possess two paralogs of the CYRI protein family, named CYRI-A and CYRI-B, which are encoded by the *FAM49A* and *FAM49B* genes, respectively. Nothing is currently known about the cellular function of CYRI-A and its relationship with CYRI-B. Through biochemical analyses and live-cell imaging, we explore the membrane kinetics and subcellular localization of CYRIs, with a strong focus on the uncharacterized CYRI-A. We implicate CYRIs as novel regulators of macropinocytosis. We then connect the function of both CYRI-A and -B to the trafficking of integrin α5β1 and their effects on cell migration, cell spreading, and cancer cell invasion. We propose that CYRI proteins act as suppressors of the RAC1–actin signaling axis at macropinocytic structures to ensure efficient completion of macropinocytosis.

## Results

### CYRI-A suppresses lamellipodial spreading and binds active RAC1

**Figure S1.**
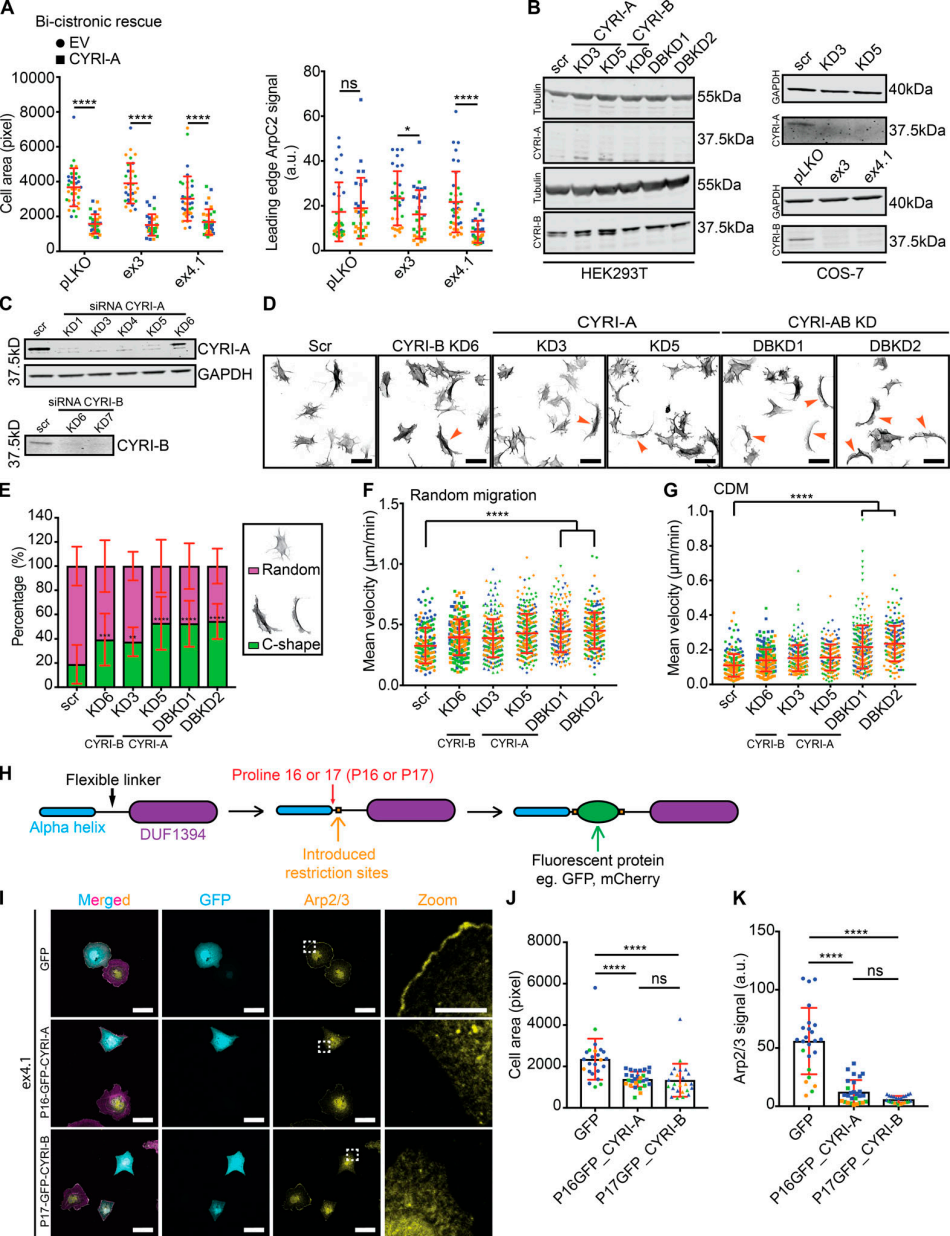
**CYRI-A and CYRI-B have complementary functions in cells. (A–G)** pLKO, control CRISPR line; ex3 and ex4.1, CYRI-B CRISPR COS-7 cell lines; scr, scramble; KD3 and KD5, CYRI-A siRNA knockdowns; KD6, CYRI-B siRNA knockdown, DBKD, KD3 + KD6, or KD5 + KD6. **(A)** Quantification of cell area (left) and the average Arp2/3 signal intensity around the cell perimeter in CYRI CRISPR COS-7 cells (ex3 and ex4.1) rescued with untagged bi-cistronic CYRI-A. Data from three independent experiments; statistical analysis using unpaired *t* test. Each experiment is color-coded. Mean ± SD. *, P < 0.05; ****, P < 0.0001. **(B)** Western blot of control or KD of CYRI-A and CYRI-B in HEK293T cells (left) and COS-7 (right) showing that HEK293T cells express only CYRI-B while COS-7 cells express both isoforms. Tubulin and GAPDH are loading controls. **(C)** Western blot of endogenous CYRI-A and CYRI-B in control or KD A-673 cells. GAPDH is loading control. **(D)** Representative immunofluorescence images of control scrambled (scr), single CYRI-A or CYRI-B, and CYRI-A/B DBKD in A-673 cells stained for F-actin (phalloidin). Orange arrowheads point to C-shaped cells. Scale bar = 50 µm. **(E)** Quantification of the number of C-shaped cells in D. Data from three independent experiments with at least 50 cells per experiment. One-way ANOVA with Tukey’s multiple comparisons. Mean ± SD. **, P < 0.01; ***, P < 0.001; ****, P < 0.0001. **(F and G)** Quantification of the mean velocity of control, single KO, and CYRI-A/B DBKD A-673 cells plated on 2D fibronectin (F) or in 3D CDM (G). Data from at least 30 cells per experiment in a total of three independent experiments. Each experiment is color-coded. One-way ANOVA with Tukey’s multiple comparison test. Mean ± SD. ****, P < 0.0001. **(H)** Schematic representation of the cloning strategy used to create the internal fluorescent tag for CYRIs. **(I)** Immunofluorescence images of control or CYRI-B CRISPR COS-7 (ex4.1) transfected with either P16-GFP-CYRI-A or P17-GFP-CYRI-B (cyan) and stained for Apr2/3 (yellow) and F-actin (magenta). Scale bar = 20 µm for full-size image or 10 µm for zoom. **(J and K)** Quantification of the cell area and the average Arp2/3 signal at the cell periphery of CYRI-B CRISPR COS-7 cells (ex4.1) expressing either GFP control or the internal-GFP tagged CYRI constructs. Data from at least 20 random fields in a total of three independent experiments. Each experiment is color-coded. One-way ANOVA with Tukey’s multiple comparison test. ns = P > 0.05; * P < 0.05; ****, P < 0.0001.

**Figure 1.**
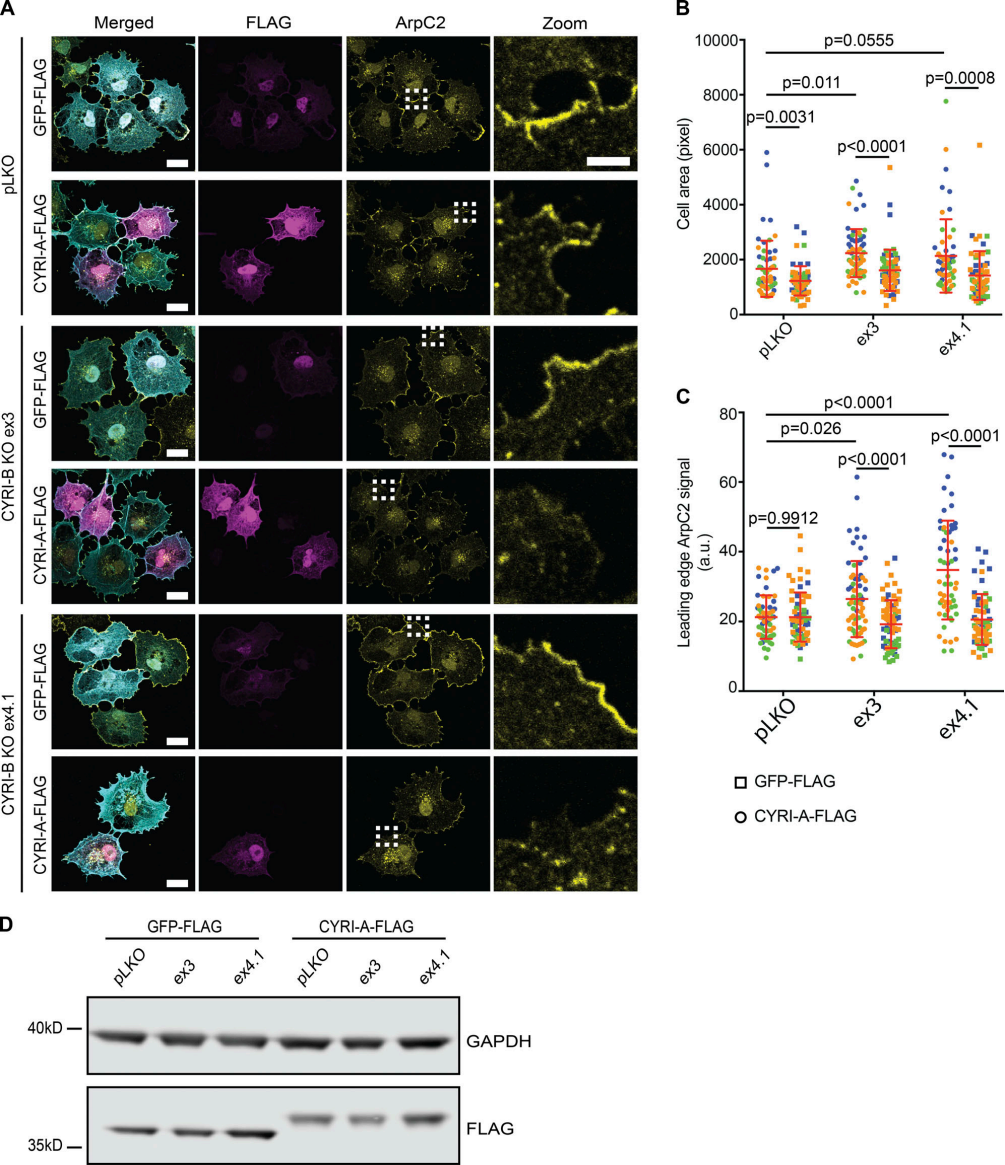
**CYRI-A suppresses cell spreading and leading-edge Arp2/3 complex recruitment.(A)** Immunofluorescence images of COS-7 pLKO (control) and CYRI-B CRISPR KO lines (ex3 or ex4.1) expressing either GFP-FLAG or CYRI-A-FLAG vector and stained for Arp2/3 complex (anti-ArpC2, yellow), FLAG-tag (magenta), and F-actin (phalloidin, cyan; scale bar = 20 µm). Dotted square denotes zooms (scale bar = 5 µm). **(B and C)** Cell spread area and Arp2/3 signal at the leading edge in COS-7 cells expressing either GFP-FLAG (squares) or CYRI-A-FLAG (circles). Data from at least 10 cells per experiment and three independent experiments, each colored separately in blue, orange, and green. Statistical analyses using two-tailed unpaired *t* test. Mean ± SD. **(D)** Representative Western blot showing relative expression of GFP-FLAG and CYRI-A-FLAG in COS-7 detected with anti-FLAG antibody. GAPDH as loading control.

While CYRI-B has been characterized as a RAC1-binding protein that restricts lamellipodia, the role of CYRI-A is unknown. Deletion of CYRI-B promotes a broad lamellipodia phenotype with enrichment of the Scar/WAVE complex at the leading edge (Fort et al., 2018; Yuki et al., 2019). Our previous study of CYRI-B used COS-7 cells, which express only very low levels of CYRI-A (Fort et al., 2018; Fig. S1). To query whether CYRI-A expression could rescue the cell-spreading phenotype caused by depletion of CYRI-B, we expressed either a FLAG-tagged GFP (control) or CYRI-A-FLAG construct (Fig. 1 A) in control or CYRI-B knockout (KO) COS-7 cells ex3 and ex4.1. CYRI-B KO cells showed enhanced lamellipodia spreading (Fort et al., 2018; Fig. 1 A), which we previously showed was rescued by reexpression of CYRI-B-FLAG (Fort et al., 2018). Here, we find that CYRI-A rescues spreading area (Fig. 1 B) and Arp2/3 recruitment (Fig. 1 C). Western blot showed comparable levels of CYRI-A-FLAG and GFP-FLAG between cell lines (Fig. 1 D). Expression of untagged CYRI-A using a bi-cistronic system provided a similar rescue (Fig. S1 A). This suggests similar functions of CYRI-A and CYRI-B in controlling lamellipodia formation.

**Figure 2.**
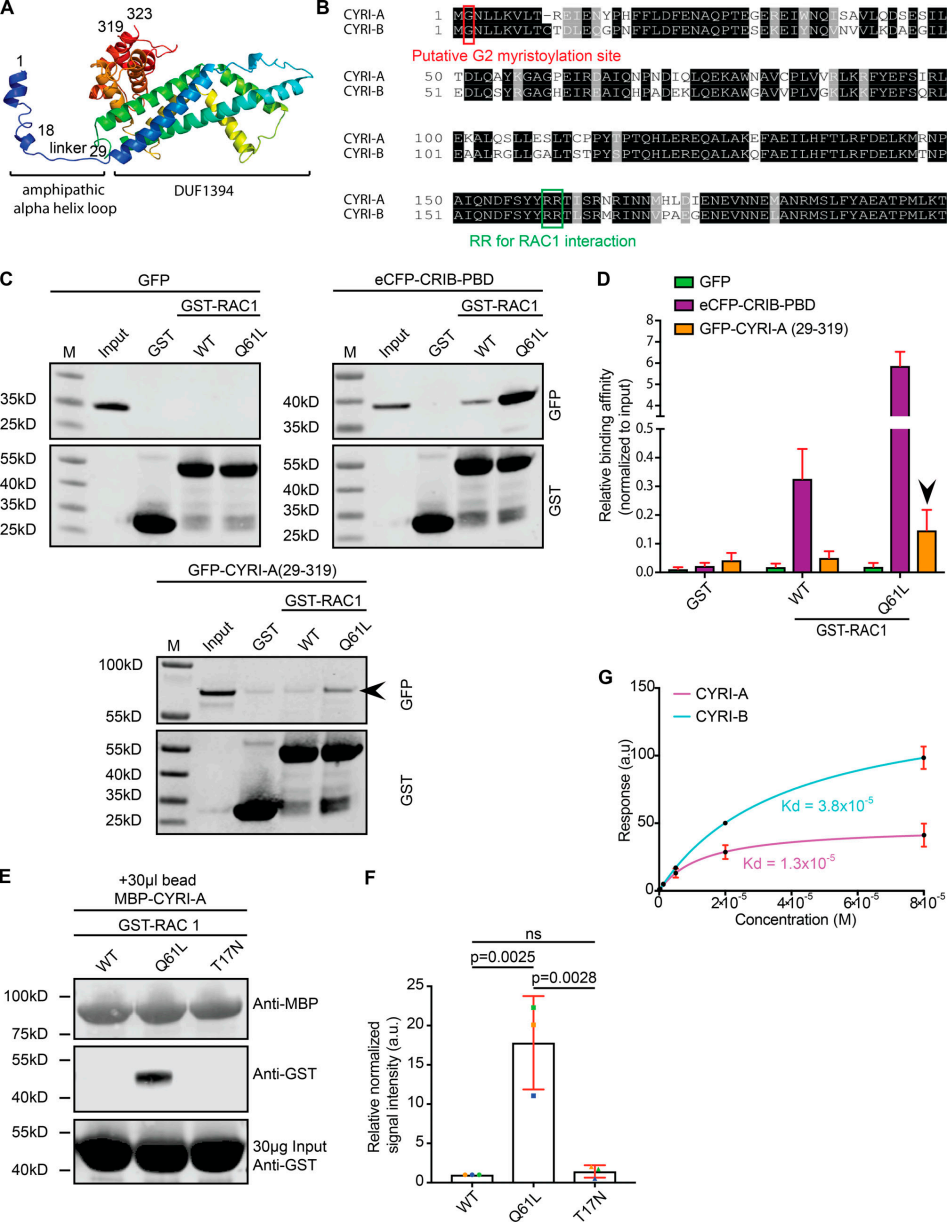
**CYRI-A is similar to CYRI-B in amino acid sequence and RAC1 binding.(A)** The molecular structure of CYRI-A as predicted by I-TASSER. The protein is composed of one N-terminal amphipathic α-helix connected to a domain of unknown function (DUF1394) via a flexible linker. Numbers represent the amino acid position. **(B)** Multiple sequence alignment using Clustal Omega of mouse CYRI-A and CYRI-B shows 80% sequence identity. Black, identical residues; gray, similar residues; red, Glycine (G2) putative myristoylation site; green, two conserved arginine residues (RR) mediating active RAC1 binding. **(C)** Western blots showing GST-Trap pulldown assay of GST or GST-RAC1 (WT or Q61L) with lysate from COS-7 cells expressing GFP (negative control), eCFP-CRIB-PBD (positive control), or GFP-CYRI-A (29–319; RBD). Probed with anti-GFP and anti-GST. Black arrowhead denotes GFP-CYRIA (29–319) and GST-RAC1 Q61L interaction. **(D)** Quantification of C from at least three independent experiments. Signals were normalized to the input. Mean ± SEM. Black arrowhead points to CYRI-A RBD and active RAC1 interaction. **(E)** Western blot showing MBP-Trap pulldown assay between MBP-CYRI-A on MBP beads interacting with GST-RAC1 WT, Q61L, or T17N (constitutively inactive). **(F)** Quantification of E from three independent experiments. Signals were normalized to MBP. Mean ± SD. Statistical analysis using one-way ANOVA with Tukey’s multiple comparisons. ns, P > 0.05. **(G)** Steady-state SPR binding curve between purified MBP-tagged full-length CYRI-A (magenta) or CYRI-B (cyan) and increasing concentrations of untagged full-length RAC1 Q61L with curve fit assuming a 1:1 binding model. *K_d_* = 1.3 × 10^−5^ (CYRI-A::RAC1 Q61L) and 3.8 × 10^−5^ (CYRI-B::RAC1 Q61L). Mean ± SD. Data from at least three independent experiments.

We next queried whether CYRI-A could interact with active RAC1. Using the I-TASSER protein prediction tool (Zhang, 2008; Roy et al., 2010; Yang and Zhang, 2015) along with the recently solved crystal structures of CYRI-B (Yelland et al., 2021; Kaplan et al., 2020), CYRI-A was predicted to contain an amphipathic N-terminal α-helix connected via a flexible linker to a bundle of 12 α-helices forming the domain of unknown function (or DUF1394 domain; Fig. 2 A). Comparing the amino acid sequence of CYRI-A and CYRI-B showed an 80% sequence identity (Fig. 2 B), including a glycine residue at the second position thought to be important for myristoylation (Fort et al., 2018) and two arginine residues at positions 159/160 (for CYRI-A) or 160/161 (for CYRI-B) that are essential for active RAC1 binding (Fort et al., 2018). Using an in vitro pulldown assay, we showed that the region of CYRI-A corresponding to the RAC1-binding domain (RBD) of CYRI-B (Fort et al., 2018; amino acids 29–319) robustly and consistently interacted with the constitutively active RAC1 Q61L (Fig. 2, C and D), but not detectably with WT or dominant-negative T17N RAC1 (Fig. 2, E and F). To interrogate whether this interaction was direct, we performed surface plasmon resonance (SPR) using purified recombinant maltose-binding protein (MBP)–tagged CYRIs immobilized on an SPR chip, reacted with soluble, untagged constitutively active RAC1 Q61L. CYRI-A interacts with active RAC1 with a dissociation constant (*K_d_*) of ∼13 µM, while for CYRI-B, the *K_d_* is ∼38 µM (Fig. 2 G). Thus CYRI-A is an active RAC1 interacting protein with a higher affinity for active RAC1 than CYRI-B.

### CYRI-A and CYRI-B cooperatively regulate cancer cell spreading and migration

**Figure 3.**
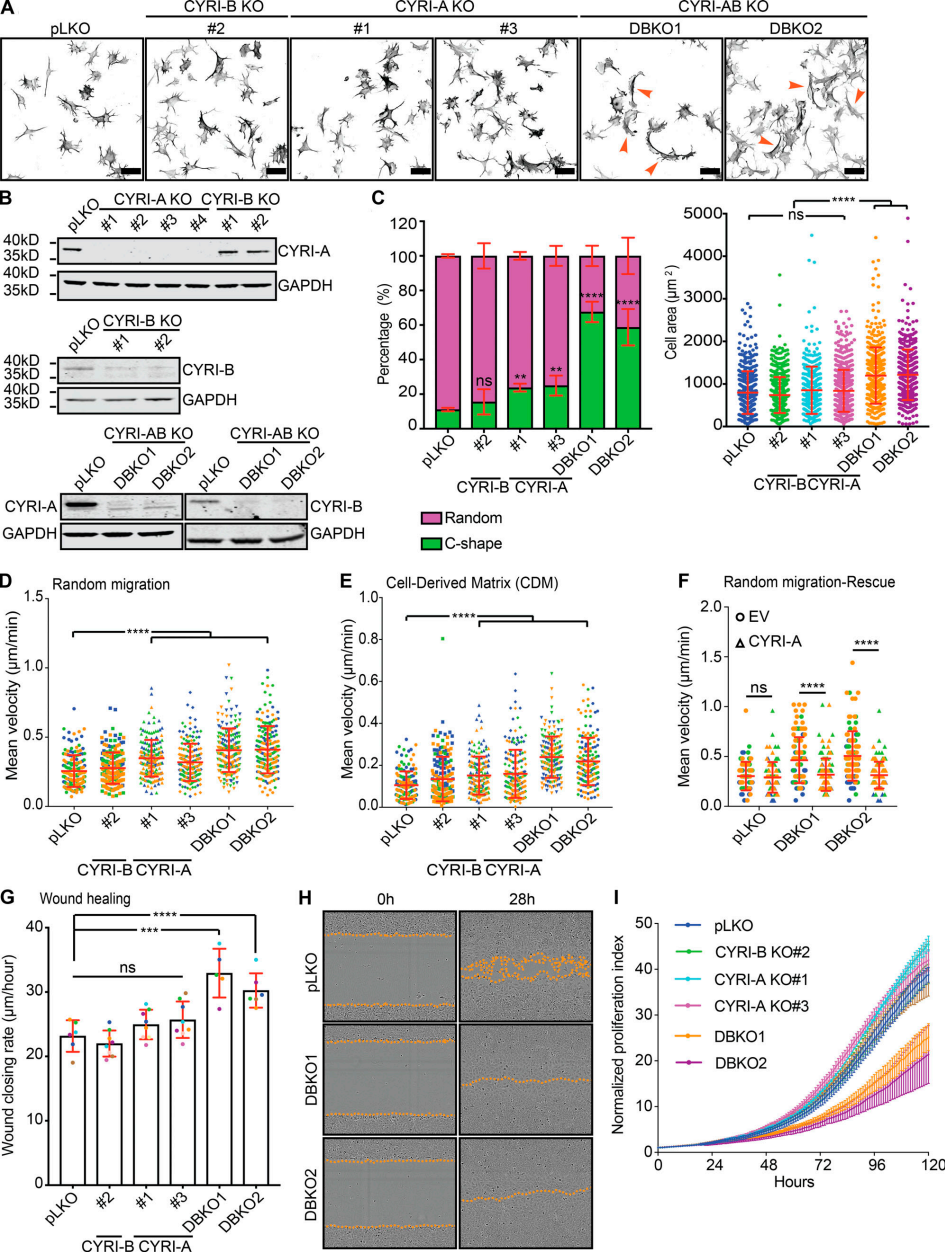
**CYRI-A and CYRI-B cooperatively regulate cell shape and migration.(A)** Immunofluorescence images of control (pLKO), single KO, and DBKO of CYRIs in A-673 cells. Cells stained for F-actin with phalloidin. Orange arrowheads indicate C-shape cells. Scale bar = 50 µm. **(B)** Western blots showing the efficiency of single KO and DBKO of CYRIs. GAPDH as loading control. **(C)** Quantification of cell shape (left) and spread area (right) of A from at least 50 cells per experiment from three independent experiments. Mean ± SD. One-way ANOVA with Tukey’s multiple comparison test. ns, P > 0.05; **, P < 0.01; ****, P < 0.0001. **(D and E)** Migration analysis of CYRI CRISPR cells on a 2D fibronectin substrate (random migration) or in 3D CDM. Data from at least 30 cells per experiment from three independent experiments. Each experiment is color-coded. Mean ± SD. One-way ANOVA with Tukey’s multiple comparison test. ****, P < 0.0001. **(F)** Reexpression of CYRI-A in DBKO A-673 cells reduces their speed to the original values but does not affect the control pLKO cells. Data from at least 30 cells per experiment from three independent experiments. Each experiment is color-coded. Mean ± SD. One-way ANOVA with Tukey’s multiple comparison test. ns, P > 0.05; ****, P < 0.0001. **(G and H)** Wound healing assay comparing the control pLKO, single KO, and DBKO CYRI cells. Data from at least four independent experiments. Each experiment is color-coded. Mean ± SD. One-way ANOVA with Tukey’s multiple comparison test. ns, P > 0.05; ***, P < 0.001; ****, P < 0.0001. Orange dotted lines highlight the edge of the cell monolayer. **(I)** Proliferation assay using the Incucyte system of control pLKO, single KO, and DBKO CYRIs in A-673 cells. Data from at least three independent experiments. Mean ± SEM.

CYRI-A is expressed at a low to undetectable level in some cell lines, including HEK293T and COS-7 (Fig. S1 B), so we sought to find cells that expressed both proteins to further understand CYRI-A’s role. Using the EMBL-EBI database, we identified Ewing’s sarcoma cell lines, and particularly A-673 cells, as expressing both isoforms at relatively comparable RNA levels. We confirmed the expression of CYRI-A and -B and the specificity of the antibody in A-673 cells using siRNA (referred to as KD or knockdown) or CRISPR-Cas9 (referred to as KO; Figs. S1 C and 3 B). Single KO of CYRI-A or CYRI-B in A-673 cells resulted in a modest but reproducible effect on the cell shape, with a 10% increase in the number of cells adopting the fast-migrating C-shape, a previously described phenotype (Yuki et al., 2019; Fort et al., 2018; Fig. 3, A and C). However, when both CYRI-A and CYRI-B were simultaneously deleted (referred to as DBKO for double knockout) we saw a large increase, with 60% of the cells adopting the C-shape morphology. We observed similar effects in cells treated with siRNA (Fig. S1, D and E). DBKO A-673 cells also showed a 30% increase in the spreading area over control pLKO (plasmid lentiviral knockdown for shRNA expression) and single KO cells. We measured the migration speed of both CRISPR or siRNA-treated cells on 2D fibronectin substrate and in 3D fibroblast cell-derived matrix (CDM; Cukierman et al., 2001; Fig. 3, D and E; and Fig. S1, F and G). In all cases, cells lacking both isoforms showed a twofold increase in their migration speed (0.5 µm/min for DBKO versus 0.2 µm/min for pLKO on 2D and 0.2 µm/min for DBKO versus 0.1 µm/min for pLKO in 3D). There were no obvious differences in motility or morphology between cells lacking just CYRI-A or CYRI-B. Reintroducing CYRI-A into the DBKO cells reduced the migration speed to basal level (Fig. 3 F). Measuring the collective migration behavior by scratch-wound assay also showed a faster wound closure rate in the DBKO cells compared with either the control pLKO or the single KO (Fig. 3, G and H). This was not due to enhanced proliferation, as DBKO cells proliferated more slowly than the other cell lines (DBKO slope, 0.11; pLKO and single KO slope, 0.15; Fig. 3 I). Overall, we find that CYRI-A and CYRI-B have a compensatory role in regulating cell shape and migration in A-673 cells.

### CYRI-A localizes to macropinocytic structures

**Figure 4.**
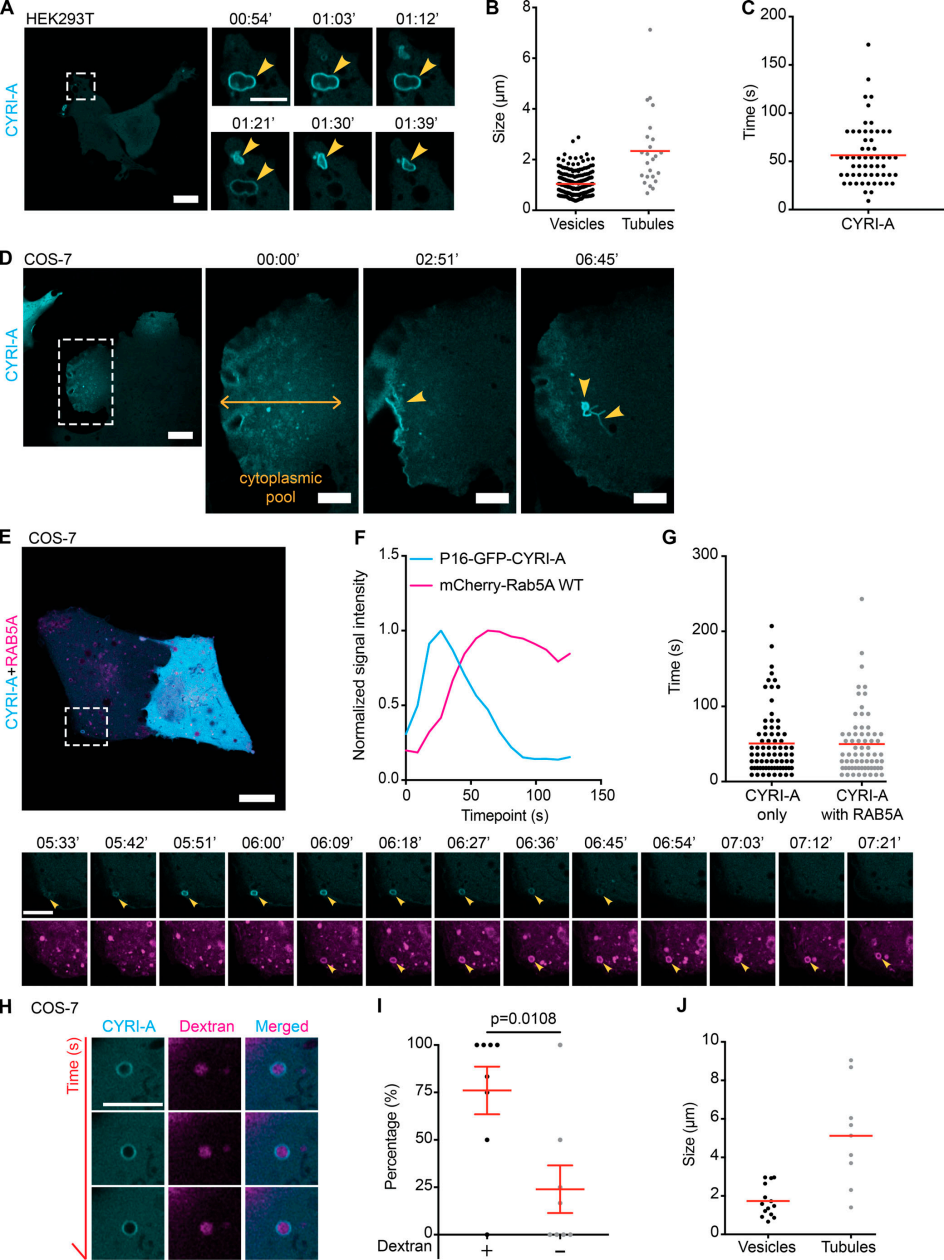
**CYRI-A localizes to large macropinocytic cup-like structures before RAB5A recruitment.** (See Video 1.) **(A–C)** P16-GFP-CYRI-A in HEK293T (scale bar = 20 µm; *n* = 204 events in 18 cells for cups/vesicles and *n* = 24 events in 10 cells for tubules) decorates structures resembling macropinocytic cups (yellow arrowheads, diameter ranging from 0.4 to 2.9 µm; scale bar = 5 µm). Tubule length 0.7–7 µm. Average lifetime of CYRI-A on cups, 50 s (*n* = 58 events in 5 cells). Red lines represent the average value. **(D)** Still images of COS-7 cells (scale bar = 20 µm) expressing P16-GFP-CYRI-A showing the diffuse pool of CYRI-A (yellow doubled arrow) near the leading edge. Dotted square denotes time sequence on the right (scale bar = 5 µm). **(E)** Time-lapse sequence of COS-7 cells expressing P16-GFP-CYRI-A (cyan) and mCherry-RAB5A (magenta). Scale bar = 20 µm (full size) or 5 µm (zoom). **(F and G)** Time sequence of CYRI-A and RAB5A recruitment to the macropinocytic cups (CYRI-A only, *n* = 75 events in 8 cells; CYRI-A with RAB5A, *n* = 68 events in 8 cells). **(H–J)** Dextran uptake assay (scale bar = 5 µm). Quantification of the percentage of CYRI-A–positive cups/vesicles containing dextran (*n* = 9 cells) and the size (cups/vesicles, *n* = 15 events in 7 cells; tubules, *n* = 10 events in 7 cells). Two-tailed unpaired *t* test.

Our results, combined with previous observations (Fort et al., 2018; Yuki et al., 2019) argued that CYRI proteins interacted directly with RAC1 and opposed its activity at the cell leading edge. However, almost nothing was known about the cellular localization or dynamics of CYRI proteins. Fluorescent tagging of CYRIs has proven difficult, as neither N- nor C-terminal tagging with GFP preserved their functions (Fort et al., 2018). In an attempt to preserve the N-terminal myristoylation and any role of the N-terminal α-helix, we inserted GFP just after the 16th proline residue of CYRI-A (P16-GFP-CYRI-A) or the 17th proline residue of CYRI-B (P17-GFP-CYRI-B; Fig. S1 H). Both constructs rescued cell spreading and Arp2/3 complex recruitment in CYRI-B CRISPR COS-7 cells, suggesting that they were functional (Fig. S1, I–K). Transiently expressing P16-GFP-CYRI-A resulted in a striking localization to vesicular and occasionally tubular structures (Fig. 4, A–D; and Video 1, clips 1 and 2). Vesicles were ∼1 µm in diameter, while tubule length varied from 2 to 8 µm. CYRI-A dynamically localized to vesicle structures, with resident times of ∼50–100 s (Fig. 4, C and G).

**Video 1.** **CYRI-A localizes to macropinocytic cup-like structures prior to RAB5A recruitment.** (Four clips; related to Fig. 4.) Clip 1 (related to Fig. 4 A): Airyscan video of HEK293T cells expressing P16-GFP-CYRI-A (cyan). Arrowheads indicate vesicles. Clip 2 (related to Fig. 4 D): Airyscan video of a COS-7 cell expressing P16-GFP-CYRI-A (cyan). Arrowheads indicate vesicles and the diffuse CYRI-A pool. Clip 3 (related to Fig. 4 E): Airyscan video of a COS-7 cell expressing P16-GFP-CYRI-A (cyan) and mCherry-RAB5A WT (magenta). Arrowheads indicate the vesicle. Clip 4 (related to Fig. 4 H): Airyscan video of a COS-7 cell expressing P16-GFP-CYRI-A (cyan) and dextran 70 kD (magenta). Arrowheads indicate the vesicle containing the dextran. Acquisition at 9 s/frame and playback at 10 fps.

**Figure S2.**
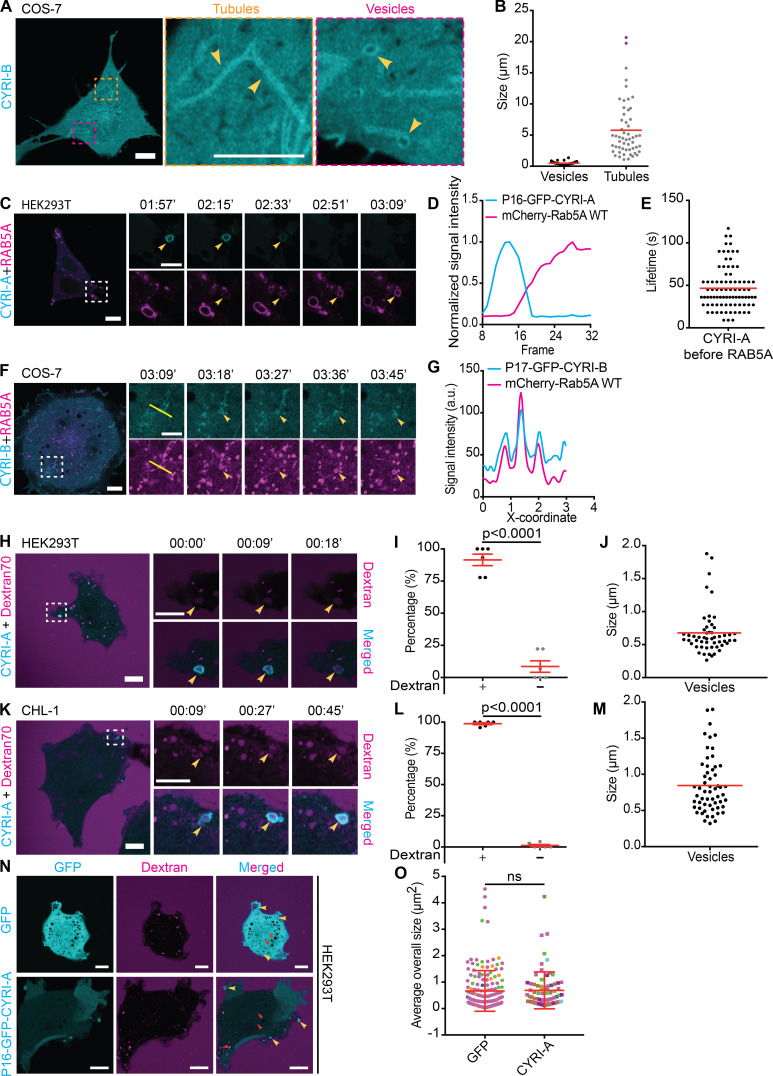
**CYRI proteins localize to macropinocytic structures prior to RAB5 arrival.** (See Video 2.) **(A and B)** Representative images of live COS-7 cells expressing P17-GFP-CYRI-B (scale bar = 10 µm). Tubular and vesicular structures are highlighted in zoomed panels, and quantification of their sizes is shown in B (vesicles, *n* = 16 events in 3 cells; tubules, *n* = 22 events in 4 cells; scale bar = 5 µm). **(C–E)** Time sequence of live HEK293T cells (scale bar = 10 µm) coexpressing P16-GFP-CYRI-A (cyan) and mCherry-RAB5A WT (magenta). Arrowhead points to vesicular structures (C; scale bar = 5 µm). The dynamics of each protein is reported by its normalized intensity plot (D) and lifetime (*n* = 84 events in 7 cells; E). **(F and G)** COS-7 cells (scale bar = 10 µm) coexpressing P17-GFP-CYRI-B (cyan) and mCherry-RAB5A WT (magenta). Time sequence corresponding to the white dotted square area is shown in the bottom panel (scale bar = 5 µm). Arrowhead points to tubular and vesicular structures, and intensity profile along the yellow line is plotted in G. **(H–M)** Time sequence images HEK293T (H) and CHL-1 cells (K) expressing P16-GFP-CYRI-A and incubated with dextran 70 kD (scale bar = 10 µm). Yellow arrowheads indicate macropinocytic events positive for both CYRI-A and dextran signals (scale bar = 5 µm). Quantification showing the majority of CYRI-A–positive vesicles are also dextran-positive in HEK293T (I; 88%, *n* = 6 cells) and CHL-1 (L; 100%, *n* = 6 cells) and their sizes (J and M; *n* = 53 events in 6 cells in HEK293T; *n* = 57 events in 6 cells in CHL-1). Red line indicates the average value. **(N and O)** HEK293T cells expressing either GFP control or P16-GFP-CYRI-A and incubated with dextran 70 kD. The size of dextran-positive vesicles in GFP (*n* = 163 events in 4 cells) is the same as that of CYRI-A–positive vesicles (*n* = 75 events in 7 cells). Events from each cell are color-coded. Unpaired *t* test. Mean ± SD.

Interestingly, CYRI-A was diffusely localized proximal to the cell leading edge in COS-7, before being recruited to the forming vesicular structures (Fig. 4 D, arrowheads). In contrast to the dynamic behavior of CYRI-A, the signal of the P17-GFP-CYRI-B construct in COS-7 cells on tubules and vesicular structures was much more stable (Fig. S2 A and Video 2, clip 1). The size of P17-GFP-CYRI-B–positive vesicles was smaller than that of P16-GFP-CYRI-A, with an average of 0.5 µm, while tubular structures were more prominent and could reach up to 20 µm (Fig. S2 B). These observations not only confirmed the ability of CYRIs to interact with the cell membrane but also suggested for the first time a potential difference in membrane kinetics between CYRI-A and CYRI-B.

**Video 2.** **CYRI proteins localize to macropinocytic structures prior to RAB5 recruitment.** (Seven clips; related to Fig. S2.) Clip 1 (related to Fig. S2 A): Airyscan video of a COS-7 cell expressing P17-GFP-CYRI-B (cyan). Clip 2 (related to Fig. S2 C): Airyscan video of a HEK293T cell expressing P16-GFP-CYRI-A (cyan) and mCherry-RAB5A WT (magenta). Arrowheads indicate the vesicles. Clip 3 (related to Fig. S2 F): Airyscan video of a COS-7 cell expressing P17-GFP-CYRI-B (cyan) and mCherry-RAB5A WT (magenta). Clip 4 (related to Fig. S2 H): Airyscan video of a CHL-1 cell expressing P16-GFP-CYRI-A (cyan) and dextran 70 kD (magenta). Clip 5 (related to Fig. S2 H): Airyscan video of a HEK293T cell expressing P16-GFP-CYRI-A (cyan) and dextran 70 kD (magenta). Clip 6 (related to Fig. S2 N): Airyscan video of a HEK293T cell expressing GFP (cyan) and dextran 70 kD (magenta). Clip 7 (related to Fig. S2 N): Airyscan video of a HEK293T cell expressing P16-GFP-CYRI-A (cyan) and dextran 70 kD (magenta). Acquisition at 9 s/frame and playback at 10 fps.

**Figure S3.**
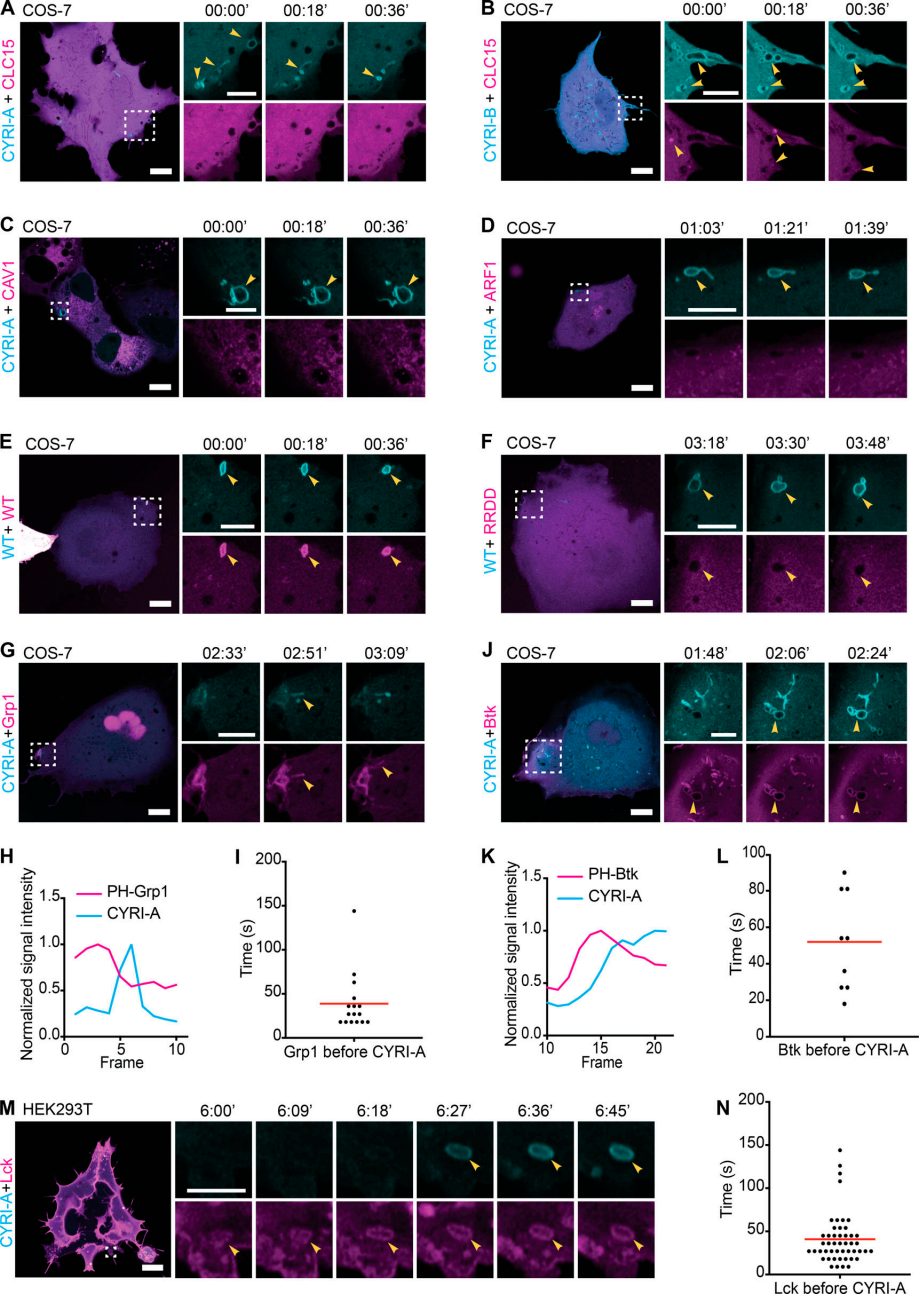
**CYRI-A-colocalizes with plasma membrane-associated nascent macropinocytic structures.** (See Video 3.) **(A–D)** Time sequence images of COS-7 cells expressing P16-GFP-CYRI-A or P17-GFP-CYRI-B (cyan) and either mCherry-tagged CLC15 (clathrin light chain 15; A and B), Caveolin-1 (C), or ARF1 (D). Scale bar = 10 µm for full-size image and 5 µm for zooms. **(E and F)** Time sequence images of live COS-7 cells coexpressing either P16-mCherry-CYRI-A WT or P16-mCherry-CYRI-A RRDD mutant (magenta) and P16-GFP-CYRI-A WT (cyan). **(G–L)** COS-7 cells coexpressing P16-GFP-CYRI-A (cyan) and two independent PIP3 reporters (magenta), PH-Grp1 (G–I) or PH-Btk (J–L; *n* = 31 events in 3 cells for Grp1; *n* = 9 events in 1 cell for Btk). Red line represents the average value. Scale bar = 10 µm for full-size image and 5 µm for zooms. **(M and N)** Time sequence images of HEK293T cells coexpressing P16-GFP-CYRI-A (cyan) and mScarlet-Lck (labeling the plasma membrane; magenta). The time Lck resides on the vesicles before CYRI-A is recruited is quantified in N (*n* = 48 events in 10 cells). Scale bar = 10 µm for full-size image and 3 µm for zooms. Red line indicates the average value.

The prominent localization of CYRIs and the size of the vesicular structures suggested a potential role in macropinocytosis. Using dual-color live imaging of COS-7 cells, P16-GFP-CYRI-A demonstrated a transient colocalization with mCherry-RAB5A (Fig. 4, E–G; and Video 1, clip 3). As the cups/vesicles formed, P16-GFP-CYRI-A was quickly recruited and remained on the cups/vesicles for ∼50 s before the RAB5A signal appeared. The gradual appearance of RAB5A coincided with the gradual loss of the CYRI-A signal, which took another 50 s (Fig. 4 G). RAB5A remained on the cups/vesicles as they were being transported further into the cells. We also verified this observation in HEK293T cells (Fig. S2, C–E; and Video 2, clip 2). On the other hand, P17-GFP-CYRI-B remained relatively evenly distributed on cups/vesicles and tubules over these timescales (Fig. S2, F and G; and Video 2, clip 3). Due to the relatively large size of many of the CYRI-A decorated structures, we speculated that they could be macropinocytic cups or macropinosomes (Canton, 2018; Swanson and Watts, 1995). Using fluorescent dextran 70 kD as a marker for fluid-phase uptake, we found that 75% of CYRI-A–positive cups/vesicles in COS-7 cells (Fig. 4, H and I; and Video 1, clip 4) and almost 100% in both HEK293T and CHL-1 cells contained dextran (Fig. S2, H–M; and Video 2, clips 4 and 5). The size of these CYRI-A–positive, dextran-positive structures (0.6–1 µm on average) was similar to Fig. 4, A–D, and consistent with macropinocytic cups or macropinosomes (Canton, 2018; Swanson and Watts, 1995; Fig. 4 J; Fig. S2, N and O; and Video 2, clips 6 and 7). We tested the colocalization of CYRI-A–decorated structures with other endocytosis pathways including clathrin-mediated endocytosis (CLC15), caveolin-mediated endocytosis (CAV1), and ARF1-dependent endocytosis (Fig. S3, A–D; and Video 3, clips 1–4) but found no clear colocalization. Thus, we found that P16-GFP-CYRI-A was recruited early in bright flashes to nascent macropinosomes, before RAB5A recruitment, suggesting a role for CYRI-A in macropinocytosis.

**Video 3.** **CYRI-A is distinct from clathrin-, caveolin-, and ARF1-mediated endocytosis and colocalizes with plasma membrane–associated macropinocytic structures.** (Nine clips; related to Fig. S3.) Clip 1 (related to Fig. S3 A): Airyscan video of a COS-7 cell expressing P16-GFP-CYRI-A (cyan) and mCherry-CLC15 (clathrin; magenta). Clip 2 (related to Fig. S3 B): Airyscan video of a COS-7 cell expressing P17-GFP-CYRI-A (cyan) and mCherry-CLC15 (clathrin; magenta). Clip 3 (related to Fig. S3 C): Airyscan video of a COS-7 cell expressing P16-GFP-CYRI-A (cyan) and mCherry-Cav1 (caveolin; magenta). Clip 4 (related to Fig. S3 D): Airyscan video of a COS-7 cell expressing P16-GFP-CYRI-A (cyan) and mCherry-ARF1 (magenta). Clip 5 (related to Fig. S3 E): Airyscan video of a COS-7 cell expressing P16-GFP-CYRI-A WT (cyan) and P16-mCherry-CYRI-A WT (magenta). Clip 6 (related to Fig. S3 F): Airyscan video of a COS-7 cell expressing P16-GFP-CYRI-A WT (cyan) and P16-mCherry-CYRI-A RRDD (magenta). Clip 7 (related to Fig. S3 G): Airyscan video of a COS-7 cell expressing P16-mCherry-CYRI-A (cyan) and GFP-GRP1 (magenta). Arrowheads indicate the vesicle. Clip 8 (related to Fig. S3 J): Airyscan video of a COS-7 cell expressing P16-mCherry-CYRI-A (cyan) and GFP-Btk1 (magenta). Arrowheads indicate the vesicle. Clip 9 (related to Fig. S3 M): Airyscan video of a HEK293T cell expressing P16-GFP-CYRI-A (cyan) and mScarlet-Lck (magenta). Arrowheads indicate the vesicle. Acquisition at 9 s/frame and playback at 10 fps.

### CYRI-A regulates macropinosome formation and forms a feedback loop with actin

**Figure 5.**
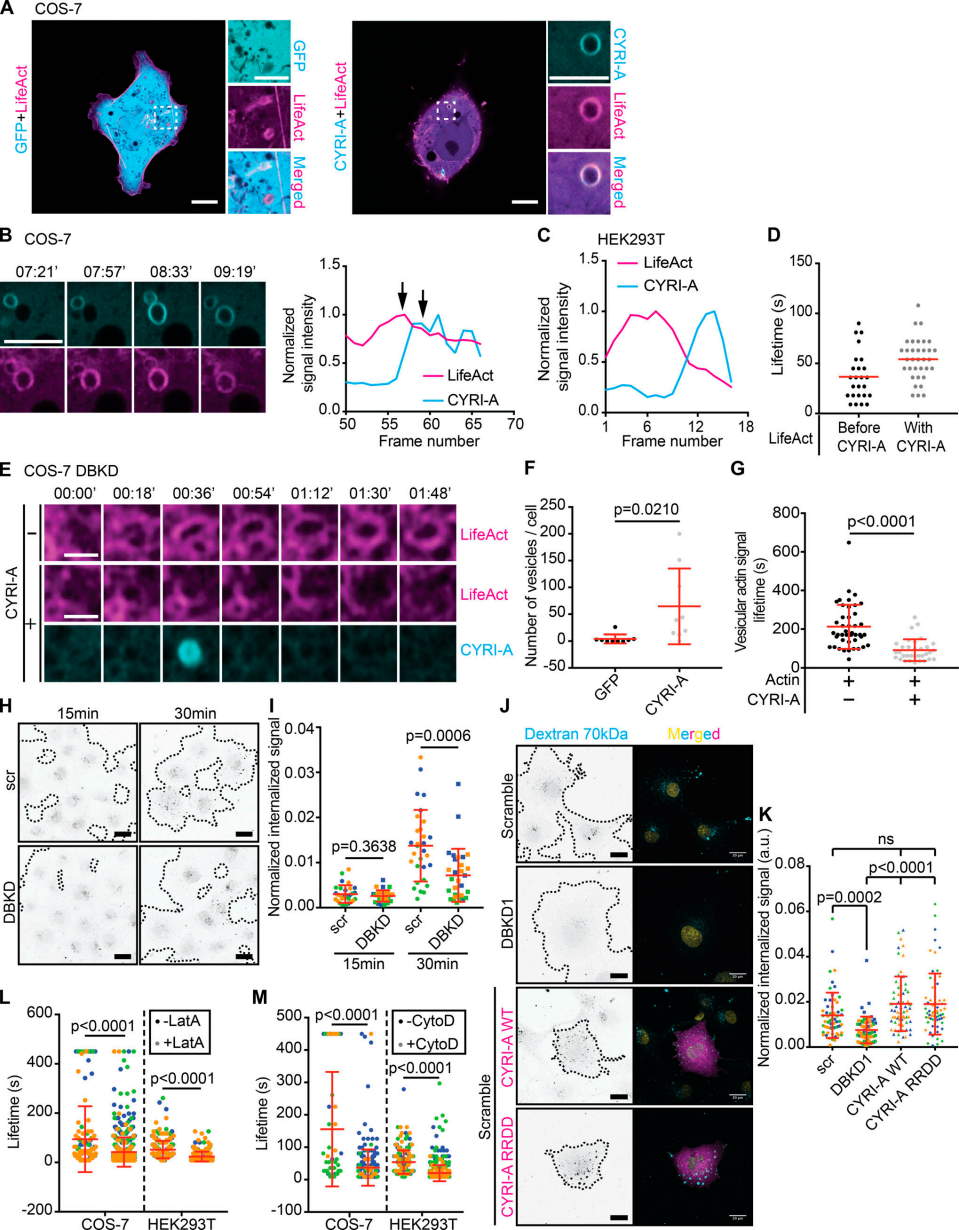
**CYRI-A regulates actin dynamics at macropinocytic structures.** (See Video 4, Video 5, and Video 6.) **(A)** Still images of COS-7 cells coexpressing either GFP (negative control) or P16-GFP-CYRI-A (cyan) and LifeAct-RFP (magenta). Scale bar = 10 µm for full-sized image and 5 µm for zooms. **(B)** Time sequence images showing the dynamics of P16-GFP-CYRI-A and actin at the macropinocytic structure in COS-7 cells. Graph shows normalized signal intensities over time. Black arrows denote peak actin and CYRI-A signals. Scale bar = 5 µm. **(C)** Normalized signal intensities over time between P16-GFP-CYRI-A and LifeAct signal in HEK293T cells. **(D)** Lifetime of actin before and after P16-GFP-CYRI-A is recruited in HEK293T cells (before CYRI-A, *n* = 25 events in 10 cells; with CYRI-A, *n* = 34 events in 10 cells). Red lines indicate the average value. **(E–G)** Lifetime of actin on macropinocytic structures ± expression of P16-GFP-CYRI-A in CYRI DBKD COS-7 cells. Scale bar = 1 µm. Number of actin-positive structures in cells ± P16-GFP-CYRI-A expression (*n* = 9 cells; F). Lifetime of the actin signal on macropinocytic structures ± P16-GFP-CYRI-A signal (actin alone, *n* = 43 events in 9 cells; actin with CYRI-A, *n* = 33 events in 8 cells). **(H)** Macropinocytosis assay in siRNA-treated COS-7 cells. Scr, scramble. Black dots are internalized dextran. Black dashed lines indicate the boundary of the cell clusters. Scale bar = 30 µm. **(I)** Macropinocytic index of H. Data are from at least 10 different fields of view per experiment from a total of three independent experiments (color-coded by experiment). Two-tailed unpaired *t* test. Mean ± SD. **(J and K)** Expression of P16-mCherry-CYRI-A in control COS-7 cells, DBKD COS-7 cells, and P16-mCherry-CYRI-A RRDD mutant (non-RAC1 binding mutant) cells showing dextran 70-kD uptake capacity of the cells. Data are from ≥10 different fields of view for a total of three independent experiments. Each experiment is color-coded. Mean ± SD. Kruskal–Wallis test with Dunn’s multiple comparison test. ns, P > 0.05. **(L and M)** Lifetime of P16-GFP-CYRI-A on macropinosomes ± 1 µM of Latrunculin A (LatA) or Cytochalasin D (CytoD) in COS-7 and HEK293T cells. At least five cells per experiment from three independent experiments (color-coded). Mean ± SD. Mann–Whitney *U* test.

RAC1-driven actin polymerization and membrane ruffling shapes early macropinocytic cups (Bloomfield and Kay, 2016; Condon et al., 2018). CYRI proteins are recruited during RAC1-mediated actin polymerization in lamellipodia (Fort et al., 2018), locally dampening down ruffles, so we hypothesized that CYRI-A could affect actin dynamics during the early stages of macropinocytosis. Cotransfecting COS-7 cells with LifeAct-RFP and P16-GFP-CYRI-A showed colocalization between actin and CYRI-A but not with GFP alone (Fig. 5 A and Video 4, clips 1 and 2). Actin accumulation at the forming macropinosome preceded CYRI-A accumulation (Fig. 5 B and Video 4, clip 3). Actin dynamics drive the early stages of macropinocytic cup formation (Yoshida et al., 2009; Mooren et al., 2012; Bloomfield and Kay, 2016). As the actin signal increased, it was sharply followed by an increase of the CYRI-A signal (Fig. 5 B, graph, COS-7 cells), and then both signals decreased, suggesting sequential recruitment. HEK293T cells showed similar dynamics (Fig. 5, C and D; and Video 4, clip 4). On average, the actin signal persisted on the structures for 36 s before the CYRI-A signal became detectable. CYRI-A and actin coincided for another 54 s on average before both decreased, similar to the lifetime of CYRI-A on these structures before RAB5A appeared (Fig. S2 E). To directly compare the temporal recruitment of CYRIs and actin during macropinocytosis, we coexpressed LifeAct-RFP and either GFP alone or P16-GFP-CYRI-A constructs in COS-7 cells depleted of both CYRI-A and CYRI-B using siRNA (DBKD for double knockdown; Fig. 5, E–G; and Video 5). As expected, DBKD COS-7 cells were flat, with relatively nondynamic lamellipodia. GFP-expressing cells formed significantly fewer macropinocytic structures (∼4 cups/vesicles/cell) compared with P16-GFP-CYRI-A rescued cells (∼65 cups/vesicles/cell). Furthermore, macropinocytic structures positive for CYRI-A had a shorter actin lifetime (∼92 s) compared with those that were negative (∼212 s). Indeed, DBKD cells accumulated significantly less 70-kD dextran than controls (Fig. 5, H and I). However, overexpressing neither P16-GFP-CYRI-A nor mutant RAC1 nonbinding P16-mCh-CYRI-A-RRDD (Yelland et al., 2021) significantly increased dextran uptake (Fig. 5, J and K), suggesting a balance of CYRI-A is required.

**Video 4.** **CYRI-A regulates actin dynamics at macropinocytic structures.** (Four clips; related to Fig. 5.) Clip 1 (related to Fig. 5 A): Airyscan video of a COS-7 cell expressing GFP (cyan) and LifeAct-RFP (magenta). Clip 2 (related to Fig. 5 A): Airyscan video of a COS-7 cell expressing P16-GFP-CYRI-A (cyan) and LifeAct-RFP (magenta). Arrows indicate the vesicles. Clip 3 (related to Fig. 5 B): Airyscan video of a zoom of a COS-7 cell expressing P16-GFP-CYRI-A (cyan) and LifeAct-RFP (magenta). Clip 4 (related to Fig. 5 C): Airyscan video of a zoom of a HEK293T cell expressing P16-GFP-CYRI-A (cyan) and LifeAct-RFP (magenta). Arrows indicate the vesicle with actin around. Acquisition at 9 s/frame and playback at 10 fps.

**Video 5.** **Knockdown of CYRI proteins affects actin dynamics at macropinocytic structures.** (Two clips; related to Fig. 5.) Clip 1 (related to Fig. 5 E): Airyscan video of a CYRI DBKD COS-7 cell expressing GFP (cyan) and LifeAct-RFP (magenta). Clip 2 (related to Fig. 5 E): Airyscan video of a CYRI DBKD COS-7 cell expressing P16-GFP-CYRI-A (cyan) and LifeAct-RFP (magenta). Acquisition at 9 s/frame and playback at 10 fps.

To query the possible actin dependence of CYRI-A localization, we treated COS-7 and HEK293T cells expressing both CYRI-A and LifeAct with 1 µM of Latrunculin A (Yarmola et al., 2000; Fig. 5 L and Video 6, clips 1 and 2). Within 30 s of addition, the actin cytoskeleton diminished greatly, but CYRI-A cups/vesicles were still frequently observed, even without any obvious membrane ruffles, and cells still showed large dextran-positive cups/vesicles (Video 6, clips 3 and 4), suggesting that even with reduced actin accumulation, CYRI was recruited to the macropinocytosis events that occurred. This suggested that CYRI-A might not directly depend on actin for its localization. However, Latrunculin A decreased the lifetime of CYRI-A at macropinocytic structures by approximately twofold in both COS-7 and HEK293T cells (Fig. 5 L and Video 6, clips 3 and 4), suggesting a positive feedback loop between actin and CYRI-A.

**Video 6.** **CYRI-A localization at macropinocytic cups is not abolished by latrunculin-A treatment.** (Four clips; related to Fig. 5.) Clip 1 (related to Fig. 5 L): Airyscan video of a COS-7 cell expressing P16-GFP-CYRI-A (cyan) and LifeAct-RFP (magenta) and treated with 1 µM of Latrunculin A (LatA). Acquisition at 30 s/frame and playback at 7 fps. Clip 2 (related to Fig. 5 L): Airyscan video of HEK293T cells expressing P16-GFP-CYRI-A (cyan) and LifeAct-RFP (magenta) and treated with 1 µM of LatA. Acquisition at 30 s/frame and playback at 7 fps. Clip 3 (related to Fig. 5 L): Airyscan video of a zoom of a COS-7 cell expressing P16-GFP-CYRI-A (cyan) and LifeAct-RFP (magenta) and treated with 0.2 mg/ml dextran 70 kD and 1 µM of LatA. Arrows indicate CYRI-A–positive vesicles that contain dextran. Acquisition at 30 s/frame and playback at 2 fps. Clip 4 (related to Fig. 5 L): Airyscan video of a zoom of a HEK293T cell expressing P16-GFP-CYRI-A (cyan) and LifeAct-RFP (magenta) and treated with 0.2 mg/ml dextran 70 kD and 1 µM of LatA. Arrows indicate CYRI-A–positive vesicles that contain dextran. Acquisition at 30 s/frame and playback at 2 fps.

Since Latrunculin A can activate the Scar/WAVE complex by an unknown mechanism (Weiner et al., 2007; Millius et al., 2009), which could potentially influence the behavior of CYRI-A, we also used Cytochalasin D (Schliwa, 1982), which showed a similar response (Fig. 5 M). Overall, these data suggest that CYRI-A is recruited to macropinocytic cups or nascent macropinosomes to RAC1 and Scar/WAVE-mediated actin assembly. CYRI-A’s lifetime, but not its recruitment, is actin dependent. This fits with our hypothesis for CYRI-A as a local inhibitor of actin assembly at nascent macropinosomes, which drives completion of macropinocytosis.

### CYRI-A regulates actin dynamics via interaction with RAC1 during macropinocytosis

**Figure 6.**
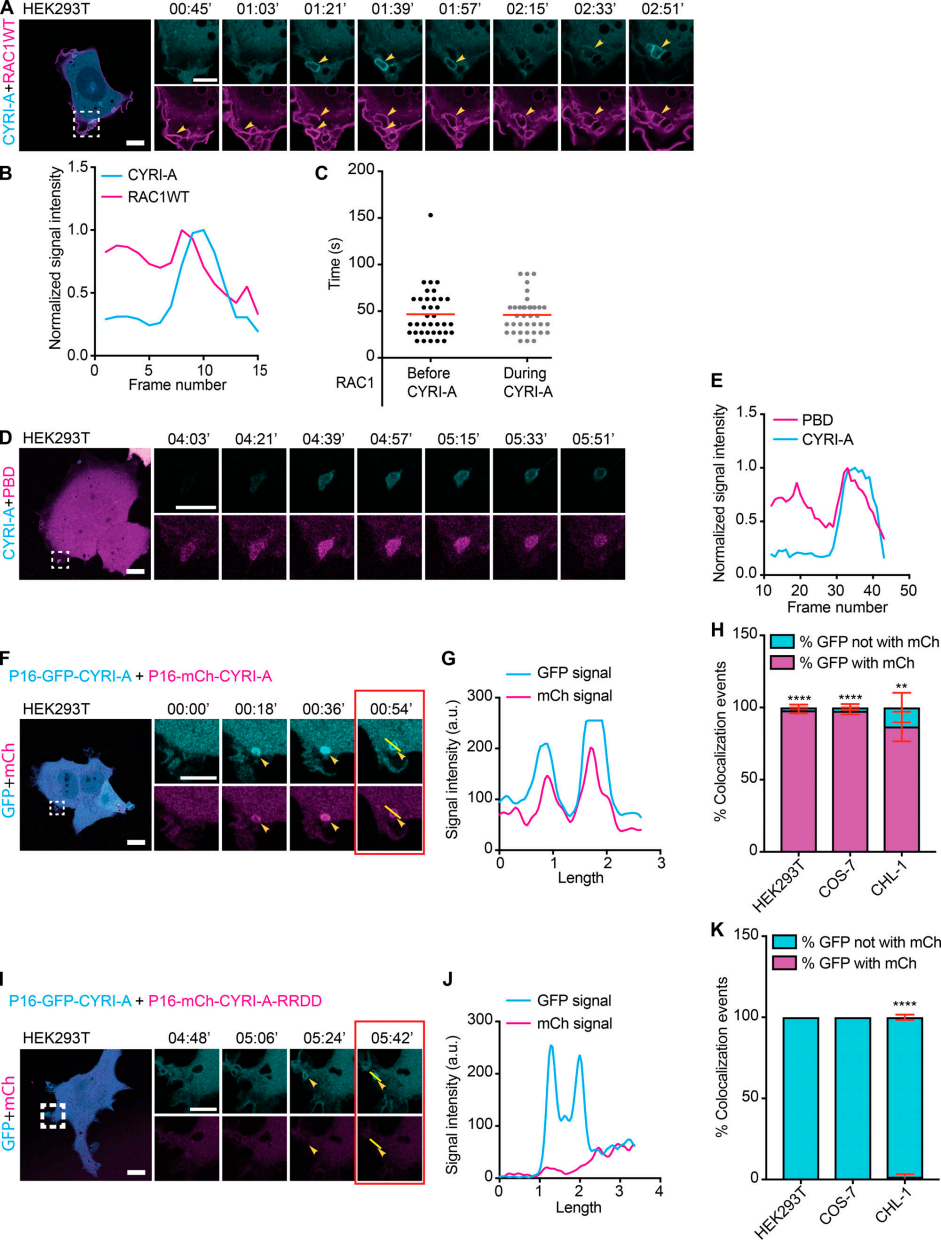
**CYRI-A is recruited to macropinocytic structures by active RAC1.** (See Video 7.) **(A–C)** Time sequence images of HEK293T cell coexpressing P16-mCherry-CYRI-A (cyan) and GFP-RAC1 WT (magenta). **(B)** Normalized signal intensity of RAC1 and CYRI-A at macropinocytic structure. **(C)** Lifetime of RAC1 signal on the macropinocytic structures (*n* = 37 events in 4 cells). Scale bar = 10 µm for full-sized image and 5 µm for zooms. **(D and E)** Time sequence images of HEK293T cell coexpressing P16-GFP-CYRI-A (cyan) and CFP-PBD (magenta). **(E)** Normalized signal intensities of CYRI-A and PBD over time. Scale bar = 10 µm for full-sized image and 5 µm for zooms. **(F–K)** Time sequence images of HEK293T cells coexpressing either WT or RRDD mutant of P16-mCherry-CYRI-A (magenta) with the WT P16-GFP-CYRI-A (cyan; F and I). Colocalization of the signals between the two WT constructs (G) and lack of colocalization between WT and mutant constructs (J). Percentage of colocalization events (*n* = 5 cells; H and K). Scale bar = 10 µm for full-sized image and 5 µm for zooms. Two-tailed unpaired *t* test. Mean ± SEM. **, P < 0.01; ****, P < 0.0001.

We next asked how dynamics of CYRI-A compared with RAC1 dynamic recruitment during macropinocytosis. Cells overexpressing GFP-RAC1 were broadly spread and displayed membrane ruffles (Fig. 6 A), with RAC1 signal enriched in ruffles and folds (Fig. 6 A and Video 7, clip 1). These ruffles folded onto themselves and formed macropinosomes (Fig. 6 A, yellow arrowhead). Importantly, the CYRI-A signal was diffuse during the very early phase where the RAC1 signal was first observed and before we observed a closed-loop structure resembling a cup. However, as the RAC1 signal gradually accumulated (for ∼50 s), it was followed by a sharp increase in the CYRI-A signal, frequently on irregularly shaped loop structures (Fig. 6, B and C). The increase in CYRI-A signal preceded the immediate drop in the RAC1 signal, and then both signals disappeared, as the macropinocytic cup progressed toward an enclosed vesicle that was carried into the cell. The timing of RAC1 accumulation matched closely to the timing of filamentous actin accumulation on these cups (Fig. 5, B–D), suggesting a mechanistic connection between CYRI-A, RAC1, and actin. We next examined the localization of active RAC1 relative to CYRI-A, using a CFP-tagged Pak-binding domain (PBD; Fig. 6, D and E; and Video 7, clip 2). Cells expressing the PBD probe showed some attenuation of macropinocytic events, but when events occurred, PBD signal accumulated as the cups matured, and before the peak of PBD, there was a sharp burst of CYRI-A signal before both dissipated. It is important to emphasize that all of these events happened locally on the macropinocytic cups and that the peak of CYRI-A and RAC1 occurred most frequently on peripherally located, irregularly shaped structures resembling macropinocytic cups and early nascent macropinosomes. We queried whether CYRI-A’s ability to bind active RAC1 was important for its recruitment to nascent macropinosomes by using the RAC1-binding defective mutant RRDD (Yelland et al., 2021). We cotransfected COS-7, HEK293T, or CHL-1 cells with the WT P16-GFP-CYRI-A and either the WT or the mutant RRDD construct of P16-mCherry-CYRI-A (Fig. 6, F–K; Video 7, clips 3 and 4; Fig. S3, E and F; and Video 3, clips 5 and 6). In all cases, GFP-tagged and mCherry-tagged WT CYRI-A colocalized at every macropinocytic event captured. In contrast, mutant CYRI-A did not colocalize with its WT counterpart on any of the macropinocytic events. Overall, these data strongly suggest that CYRI-A is locally recruited to macropinocytic structures as they form and with similar dynamics to RAC1, dependent on the RAC1 binding motif in CYRI-A.

**Video 7.** **CYRI-A recruitment coincides with and depends on active RAC1 recruitment to macropinocytic structures.** (Four clips; related to Fig. 6.) Clip 1 (related to Fig. 6 A): Airyscan video of a HEK293T cell expressing P16-mCherry-CYRI-A (cyan) and GFP-RAC1 WT (magenta). Arrowheads indicate the vesicles. Clip 2 (related to Fig. 6 D): Airyscan video of a HEK293T cell expressing P16-mCherry-CYRI-A (cyan) and CFP-PBD (magenta). Arrowheas indicate the vesicle. Clip 3 (related to Fig. 6 F): Airyscan video of a HEK293T cell expressing P16-GFP-CYRI-A WT (cyan) and P16-mCherry-CYRI-A WT (magenta). Clip 4 (related to Fig. 6 I): Airyscan video of a HEK293T cell expressing P16-GFP-CYRI-A WT (cyan) and P16-mCherry-CYRI-A RRDD (magenta). Acquisition at 9 s/frame and playback at 10 fps.

### Recruitment of CYRI-A during macropinocytosis is dependent on PI3K signaling

**Figure 7.**
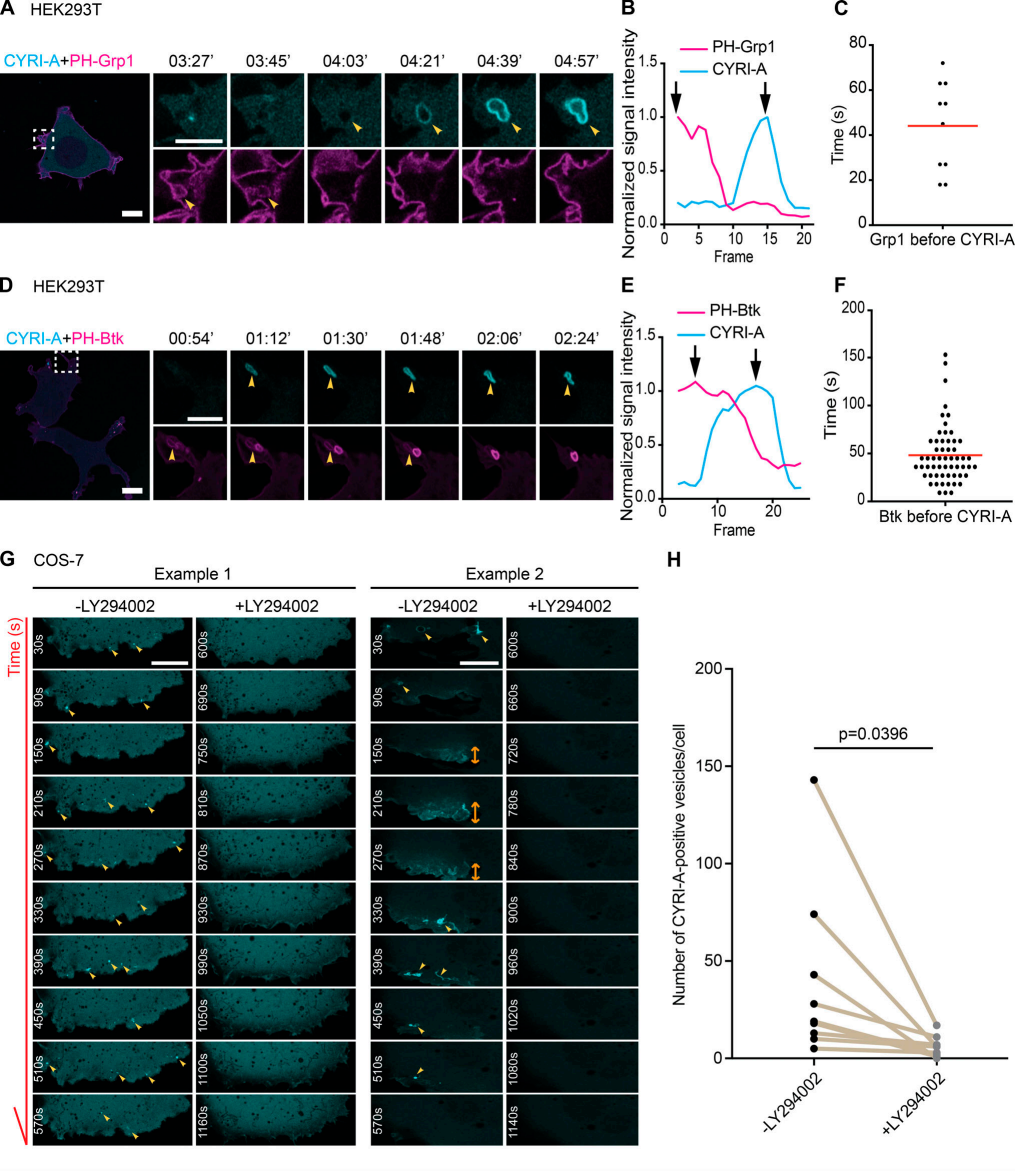
**CYRI-A’s recruitment to macropinocytic structures is dependent on PI3K signaling.** (See Video 8.) **(A–F)** HEK293T cells were cotransfected with P16-mCherry-CYRI-A (cyan) and either GFP-PH-Grp1 (magenta) or GFP-PH-Btk (magenta) as specific markers for PIP3 (A and D). Line graphs show the sequential events between PIP3 reporters and CYRI-A (B and E). Black arrows indicate the peaks of each normalized signal. Scatter plots show the average lifetime of PIP3 reporter signal before CYRI-A is recruited to macropinocytic structures (Grp1, *n* = 9 events in 3 cells; Btk1, *n* = 57 events in 6 cells). Red lines represent the average value. Scale bar = 10 µm for full-sized images and 5 µm for zooms. **(G and H)** Time sequence images showing COS-7 cells expressing P16-GFP-CYRI-A before and after the addition of 20 µM of LY294002. Quantification shows a significant decrease in the number of P16-GFP-CYRI-A–positive cups/vesicles formed upon PI3K inhibition (*n* = 9 cells). Scale bar = 10 µm. Statistical analysis using paired *t* test.

PI3K signaling plays an important role during macropinocytosis, as phosphatidylinositol (3,4,5)-trisphosphate (PIP3) production has been shown to recruit many RAC1 guanine nucleotide exchange factors (GEFs; Araki et al., 2007; Campa et al., 2015; Bloomfield and Kay, 2016; Bohdanowicz and Grinstein, 2013). We visualized PIP3 using two independent reporters, PH-Grp1 (Lai et al., 2013; Kavran et al., 1998) and PH-Btk (Araki et al., 2007; Várnai et al., 2005), along with the internally tagged CYRI-A construct in HEK293T cells. Remarkably, the PIP3 signal appeared for ∼40 s on what appeared to be irregularly shaped cups forming at the periphery of the cell and peaked before the CYRI-A signal slowly appeared (Fig. 7, A–F; and Video 8, clips 1 and 2). COS-7 cells were similar, although PIP3 reporters sometimes also localized to tubules clumping together (Fig. S3, G–L; and Video 3, clips 7 and 8). Our observations fit nicely with the consensus model where PIP3 recruited RAC1 GEFs, which activated RAC1 and drove actin polymerization (Buckley and King, 2017; Swanson and Watts, 1995; Bloomfield and Kay, 2016), and the recruitment of CYRI-A could dampen down this signaling pathway. Blocking PI3K activity using 20 µM LY294002 almost completely disrupted the recruitment of CYRI-A to the cups/vesicles in COS-7 cells (Fig. 7, G and H; and Video 8, clips 3 and 4). It is interesting to note that cells treated with LY294002 still displayed low levels of membrane ruffles (Araki et al., 1996), but both the diffused and localized recruitment of CYRI-A was completely abolished. This fits in with a previous observation that LY294002 blocked the resolution of macropinocytic cups (Araki et al., 1996). Interestingly, mScarlet-Lck (Chertkova et al., 2020 *Preprint*) marked the plasma membrane, which invaginated for 50 s before CYRI-A was recruited (Fig. S3, M and N; and Video 3, clip 9). The CYRI-A signal appeared coincidently with the loss of plasma membrane identity of the structures, indicating cup closure and detachment of the vesicles into the cytoplasm. This suggests that the CYRI-A signal coincides with the transition from a macropinocytic cup at the plasma membrane into an enclosed vesicle that is then transported into the cell. Overall, the function of CYRI-A is strongly dependent on PI3K-PIP3 activity, which may be due to the central role of PI3K-PIP3 in macropinocytic cup formation.

**Video 8.** **CYRI-A recruitment to macropinocytic structures depends on PI3-kinase.** (Four clips; related to Fig. 7.) Clip 1 (related to Fig. 7 A): Airyscan video of a HEK293T cell expressing P16-mCherry-CYRI-A (cyan) and GFP-GRP1 (magenta). Arrowheads indicate the vesicle. Acquisition at 9 s/frame and playback at 10 fps. Clip 2 (related to Fig. 7 D): Airyscan video of a HEK293T cell expressing P16-mCherry-CYRI-A (cyan) and GFP-Btk (magenta). Arrowheads indicate the vesicle. Acquisition at 9 s/frame and playback at 10 fps. Clip 3 (related to Fig. 7 G): Example 1 of an Airyscan video of a COS-7 cell expressing P16-mCherry-CYRI-A (cyan) and treated with 20 µM LY294002 (as indicated). Arrowheads indicate the vesicles. Acquisition at 30 s/frame and playback at 7 fps. Clip 4 (related to Fig. 7 G): Example 2 of an Airyscan video of a COS-7 cell expressing P16-mCherry-CYRI-A (cyan) and treated with 20 µM LY294002 (as indicated). Arrowheads indicate the vesicles. Acquisition at 30 s/frame and playback at 7 fps.

### CYRI proteins affect the trafficking of integrin α5β1

**Figure 8.**
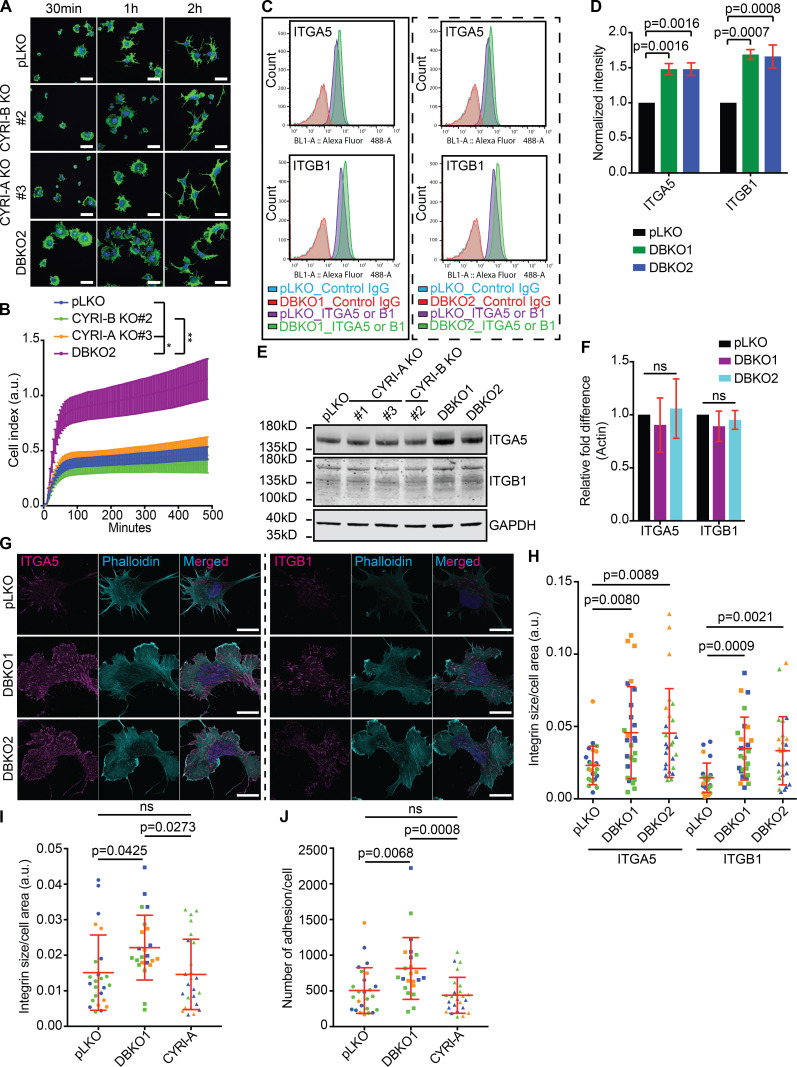
**CYRI-A and CYRI-B cooperatively regulate matrix attachment and surface expression of integrin α5β1 in A-673 cells.(A and B)** Representative immunofluorescence images of A-673 cells comparing the spreading propensity between the control pLKO and the CYRI DBKO cells on fibronectin (A). Scale bar = 40 µm. Graph shows a time course of cell–matrix attachment for control pLKO (blue), the single KO (green and orange), and the DBKO cells. **(C and D)** Flow cytometry analysis (C) and quantification (D) of the surface expression of integrin α5β1 in A-673 cells between control pLKO and the DBKO cells (DBKO1 and DBKO2). **(E and F)** Representative Western blot of the total level of integrin α5β1 in DBKO cells compared with control pLKO or single KO cells (E). qPCR analysis for gene expression of integrin α5β1 in pLKO and the DBKO cells (F). **(G and H)** Immunofluorescence images of the surface level of integrin α5β1 between the control pLKO and DBKO cells (#1 and #2; G). Scale bar = 10 µm. The quantification is shown in H. Data from ≥10 cells per experiment in a total of three independent experiments. Each experiment is color-coded. **(I and J)** Effect of CYRI-A expression in DBKO cells on area and number of integrin adhesion sites. Data from ≥10 cells per experiment in a total of three independent experiments. Each experiment is color-coded. For all graphs, mean ± SD. Statistical analysis using one-way ANOVA with Tukey’s multiple comparison test.

**Figure S4.**
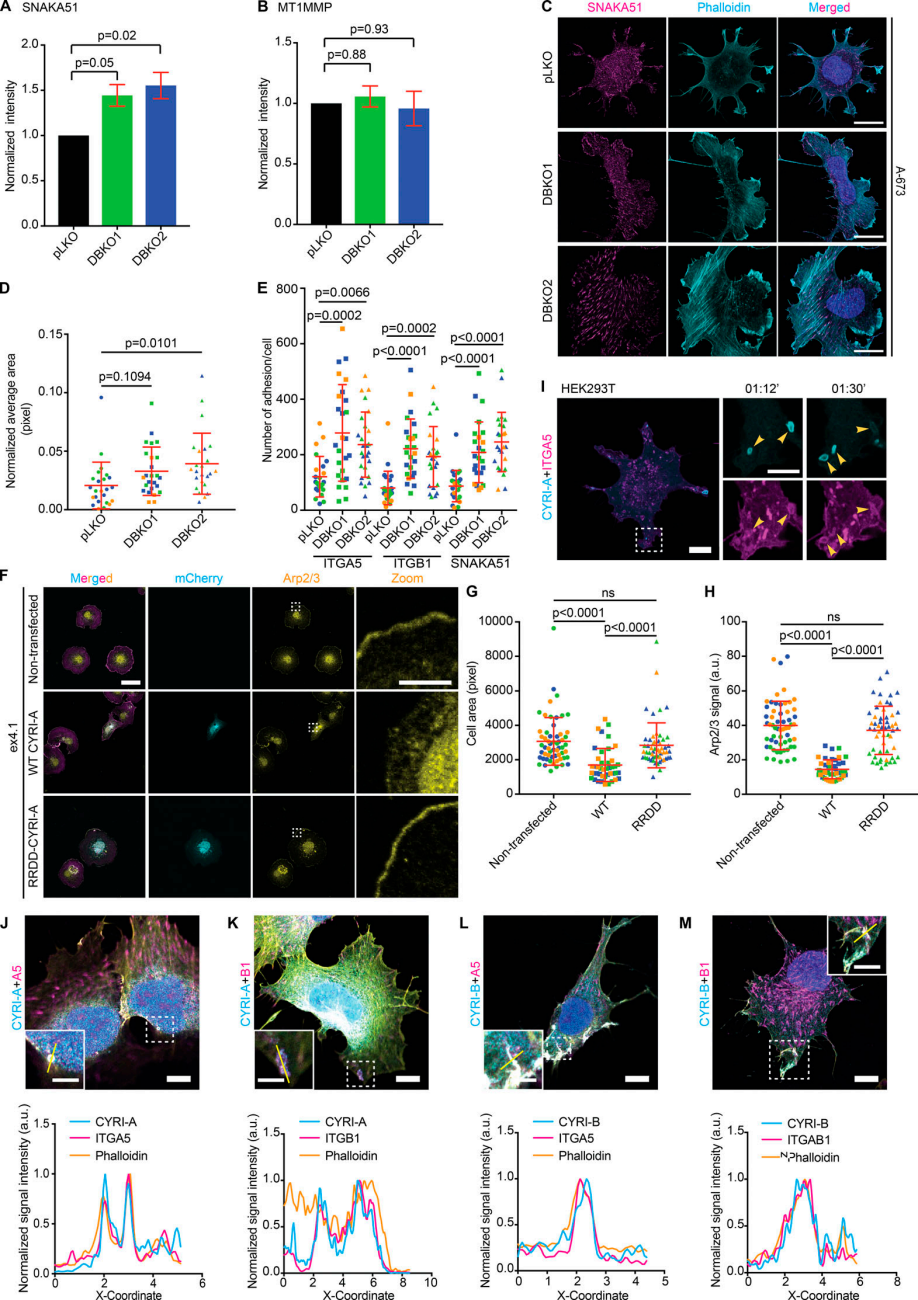
**CYRIs affect integrin α5β1 trafficking.** (See Video 9.) **(A and B)** Flow cytometry analysis of surface expression of active integrin α5, detected using the SNAKA51 antibody (A) and MT1MMP (B) comparing the control pLKO and CYRI-A/B DBKO A-673 cells. Data from three independent experiments. Statistical analysis using one-way ANOVA with Tukey’s multiple comparison test. **(C–E)** Immunofluorescence images of the control pLKO and DBKO A-673 cells stained for active integrin α5 (magenta) and actin (cyan). The average area of integrin clusters or the number of clusters per cell are quantified in D and E. Data from three independent experiments with at least 10 cells per experiment. Each experiment is color-coded. Mean ± SD. Statistical analysis use one-way ANOVA with Tukey’s multiple comparison test. Scale bars = 20 µm. **(F–H)** Non-RAC1-binding mutant P16-mCherry-CYRI-A RRDD does not rescue the spreading phenotype of CYRI-B KO COS-7. Quantification of the cell spread area (G) and the Arp2/3 signal accumulating at the cell periphery (H) show that WT CYRI-A rescued these phenotypes in CYRI-B KO COS-7, while RRDD mutant did not. Data from at least 10 random fields of view in a total of three independent experiments. Each experiment is color-coded. Statistical analysis using one-way ANOVA with Tukey’s multiple comparison test. Mean ± SD. ns, P > 0.05. **(I)** Time sequence images of HEK293T cells coexpressing P16-GFP-CYRI-A (cyan) and mApple-integrin α5 (magenta) showing integrin α5 signal present on CYRI-A–positive vesicles. Scale bar = 10 µm for full-sized image and 5 µm for zooms. **(J–M)** Immunofluorescence images of endogenous integrins α5 and β1 in A-673 cells with the P16-GFP-CYRI-A or P17-GFP-CYRI-B constructs along with actin (yellow). Graphs show the colocalization of CYRI-A, integrins, and filamentous actin (phalloidin) on the vesicles. Scale bars = 10 µm. In C, F, and J–M: DAPI for DNA.

One of the major cargoes of macropinocytic uptake is integrins (Caswell et al., 2009; Gu et al., 2011; De Franceschi et al., 2015). Furthermore, the broad lamellipodial phenotype of CYRI DBKO cells suggested a possible enhancement of integrin-based adhesion. We queried whether CYRI proteins could impact adhesion and integrin trafficking. using the xCELLigence adhesion assay based on impedance to show that CYRI DBKO A-673 cells spread approximately twofold more efficiently than single KOs or controls (Fig. 8, A and B). Flow cytometry analysis revealed an almost 50% increase in the surface expression of integrin α5 and β1 in two independent CYRI DBKO A-673 cell lines compared with control pLKO (Fig. 8, C and D). Western blotting showed a minor but consistent increase of the total level of integrin α5 and β1, but quantitative real-time PCR (qPCR) analysis showed no obvious change in their mRNA levels (Fig. 8, E and F). We detected a similar increase for the active integrin α5 using the SNAKA51 antibody with flow cytometry, but no change for the level of the membrane metalloprotease MT1MMP (Fig. S4, A and B). Immunofluorescence analysis revealed a significant increase in both the area of adhesion sites as well as their number per cell in the CYRI DBKO cells compared with the controls (Fig. 8, G and H; and Fig. S4, C–E). Overexpression of P16-GFP-CYRI-A in the DBKO cells rescued these phenotypes (Fig. 8, I and J), while the non-RAC1-binding mutant failed to rescue the phenotype of CYRI-B KO in COS-7 cells (Fig. S4, F–H; and Video 9, clip 3). Overall, we find that loss of both CYRI isoforms in A-673 cells leads to the enhanced display of surface integrins, suggesting a defect in their intracellular trafficking.

**Figure 9.**
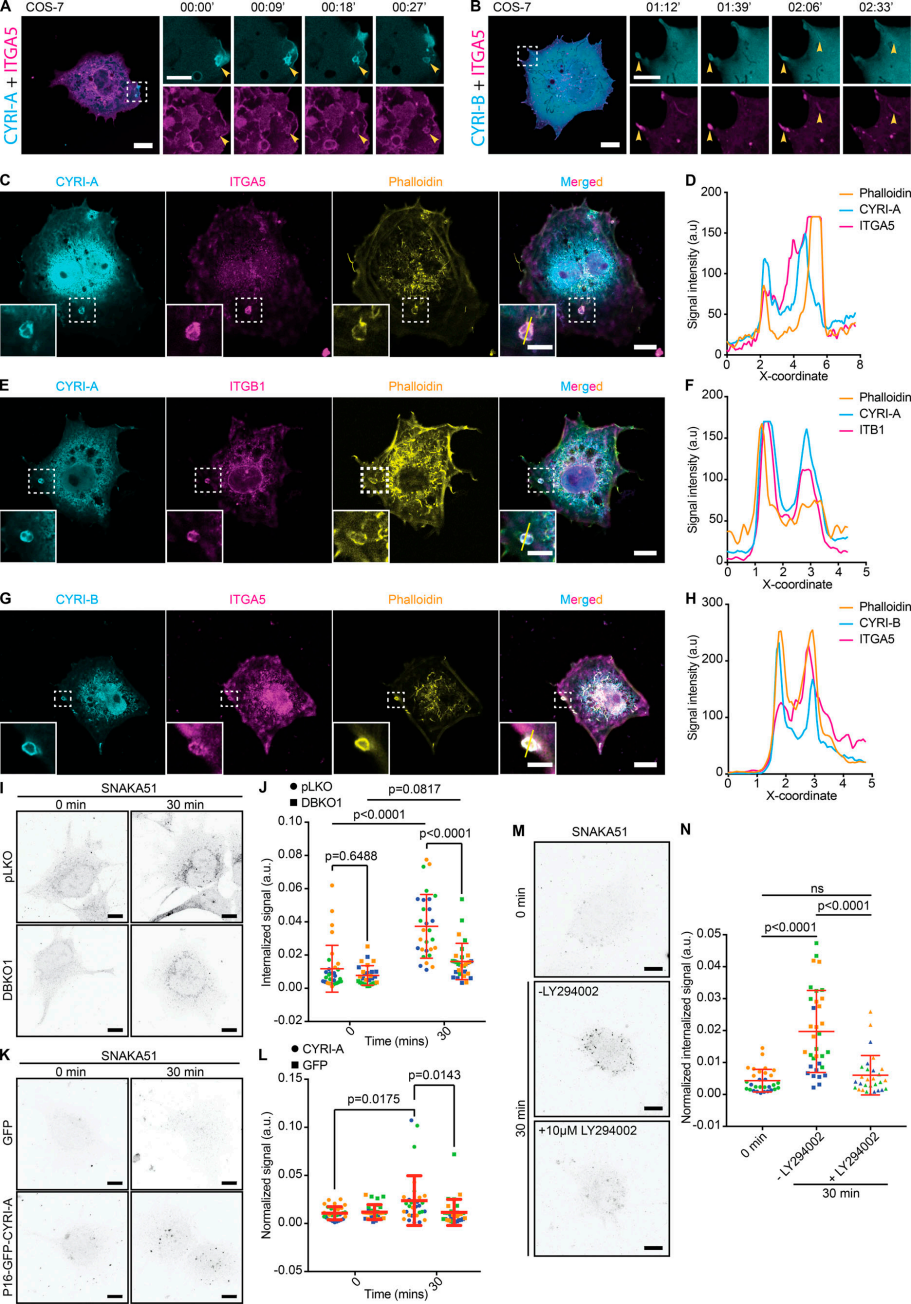
**CYRI-A and CYRI-B cooperatively regulate the internalization of integrin α5β1.** (See Video 9.) **(A and B)** Time sequence images of COS-7 cells coexpressing either P16-GFP-CYRI-A or P17-GFP-CYRI-B and mApple-tagged integrin α5. Scale bar = 10 µm for full-sized image and 5 µm for zooms. **(C–H)** Colocalization analysis of P16-GFP-CYRI-A or P17-GFP-CYRI-B (cyan) with endogenous integrin α5 or β1 (magenta) and actin (orange) in COS-7 cells. Right panels (D, F, and H) are intensity graphs showing the colocalization of all three normalized signals at the cups/vesicles. Scale bar = 10 µm for full-sized image and 5 µm for zooms. **(I and J)** Internalization assay of A-673 cells for active integrin α5 (SNAKA51, cyan) after 0- or 30-min incubation. **(I)** Representative images of the internalized signal of active integrin α5 (SNAKA51, black dots). **(J)** Quantification of the area of the internalized signals. Data from three independent experiments of at least 10 cells per experiment. Each experiment is color-coded. Statistical analysis using one-way ANOVA with Tukey’s multiple comparison test. Mean ± SD. Scale bar = 10 µm. **(K and L)** Reexpression of P16-GFP-CYRI-A in CYRI DBKO A-673 cells rescued the internalization defect in these cells compared to cells expressing only GFP. **(L)** Quantification of K. Data from three independent experiments of at least 10 cells per experiment. Each experiment is color-coded. Statistical analysis using one-way ANOVA with Tukey’s multiple comparison test. Mean ± SD. Scale bar = 10 µm. **(M and N)** CYRI DBKO A-673 reexpressing P16-GFP-CYRI-A treated with 10 µM LY294002 show reduction in the internalized active integrin α5 signal compared the untreated cells. **(N)** Quantification of at least 10 cells per experiment in a total of three independent experiments. Each experiment is color-coded. Statistical analysis using one-way ANOVA with Tukey’s multiple comparison test. Mean ± SD. Scale bar = 10 µm. ns, P > 0.05.

**Video 9.** **Integrins colocalize with CYRI-A at macropinocytic structures.** (Three clips; related to Fig. 9.) Clip 1 (related to Fig. 9 A): Airyscan video of a COS-7 cell expressing P16-GFP-CYRI-A (cyan) and mApple-ITGA5 (magenta). Arrowheads indicate the vesicles. Clip 2 (related to Fig. 9 B): Airyscan video of a COS-7 cell expressing P17-GFP-CYRI-B (cyan) and mApple-ITGA5 (magenta). Arrowheads indicate the vesicles. Clip 3 (related to Fig. S4 F): Airyscan video of a HEK293T cell expressing P16-GFP-CYRI-A (cyan) and mApple-ITGA5 (magenta). Arrowheads indicate the vesicles. Acquisition at 9 s/frame and playback at 10 fps.

### Integrin α5 and β1 are localized to CYRI-positive cups and vesicles

Since CYRIs are involved in macropinosome formation and macropinocytosis is one of the major effectors of bulk internalization of integrins (Caswell et al., 2009; Gu et al., 2011; De Franceschi et al., 2015), we next asked whether integrins colocalized with CYRI proteins at these structures. We first coexpressed an mApple-tagged integrin α5 construct along with the P16-GFP-CYRI-A or P17-GFP-CYRI-B in COS-7 cells (Fig. 9, A and B; and Video 9, clips 1 and 2) or HEK293T cells (Fig. S4 F). Integrin α5 decorated many intracellular cups/vesicles, including CYRI-A–positive macropinocytic cups/vesicles formed near the edge of the cells (Fig. 9 A). CYRI-B also decorated tubules emanating from the tips of filopodia-like protrusions (Fig. 9 B). Integrins also colocalized with CYRI-A– and CYRI-B–positive macropinocytic structures in COS-7 (Fig. 9, C–H) as well as in the DBKO A-673 cells (Fig. S4, J–M).

Increased surface presentation of integrin α5 and β1 suggested a potential defect in internalization, perhaps due to the requirement for CYRI proteins in the resolution of macropinocytic structures. To address this, we performed an internalization assay of the active integrin α5. Control cells showed a significant increase in internalized signal after 30 min (Fig. 9, I and J). Expression of the P16-GFP-CYRI-A in CYRI DBKO A-673 cells rescued their integrin internalization defect, while treating with LY294002 significantly decreased the internalized signal (Fig. 9, K–N). This strongly suggests the role of macropinocytosis in contributing to integrin internalization in these cells. Overall, our data support a novel function of CYRIs in cooperatively regulating the trafficking of integrin α5β1 through the enhancement of actin dynamics during macropinocytosis to allow the efficient internalization of surface integrins.

### CYRI DBKO A-673 cells are more invasive and resistant to anoikis due to the increased level of surface integrins

**Figure 10.**
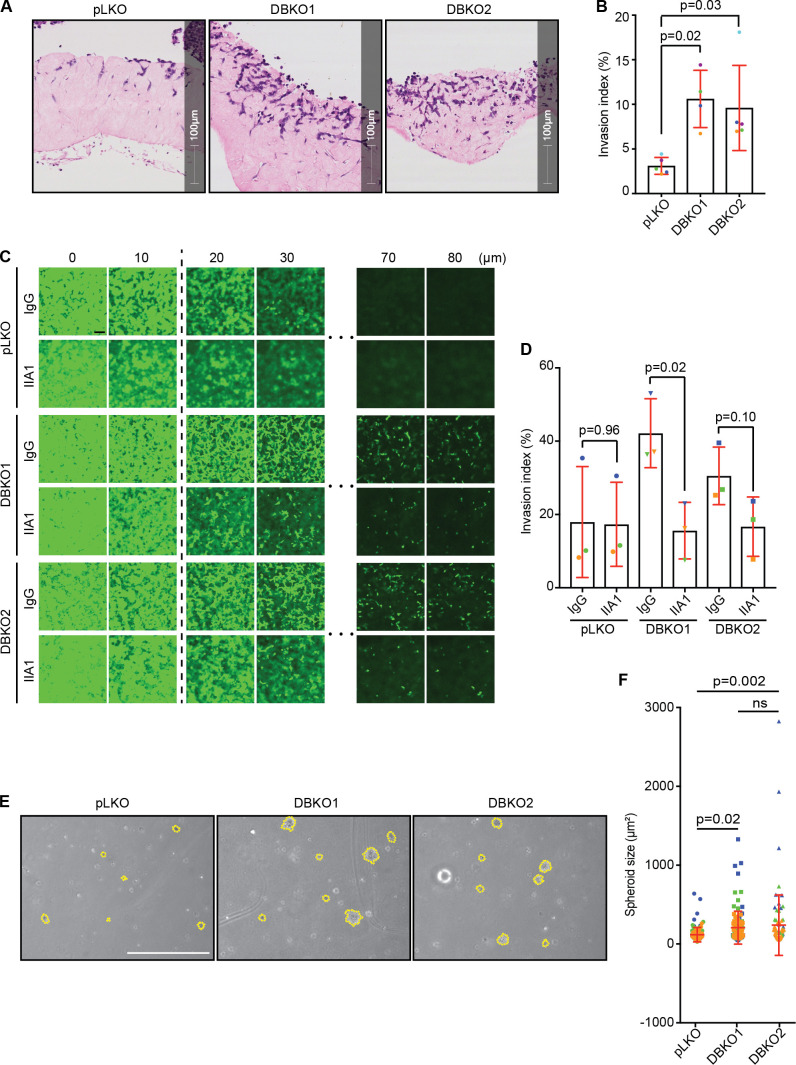
**CYRI proteins affect the invasion ability of cancer cells through integrins.(A and B)** Organotypic assay of control pLKO versus CYRI DBKO A-673 cells stained with hematoxylin and eosin. **(B)** Quantification of the invasion index (the area invaded by cells divided by the total area of the plug). Data from at least three independent experiments. Each experiment is color-coded. Mean ± SD. Statistical analysis using ANOVA with Tukey’s multiple comparison test. **(C and D)** Inverted invasion assay comparing the invasion capacity between the control pLKO and the CYRI DBKO A-673 cells in the presence or absence of the integrin α5β1-blocking antibody IIA1. Scale bar = 100 µm. **(D)** The graph shows the quantification of the invasion index (the area covered by cells beyond 10 µm into the plug). Data from at least three independent experiments. Each experiment is color-coded. Mean ± SD. Statistical analysis using two-tailed unpaired *t* test. **(E and F)** Phase-contrast images of the soft agar assay comparing the capacity for anchorage-independent growth of the control pLKO and the CYRI DBKO A-673 cells. The size of the spheroids is quantified in F. Scale bar = 5 cm. Data from three independent experiments. Each experiment is color-coded. Mean ± SD. Statistical analysis using ANOVA with Tukey’s multiple comparison test. ns, P > 0.05.

**Figure S5.**
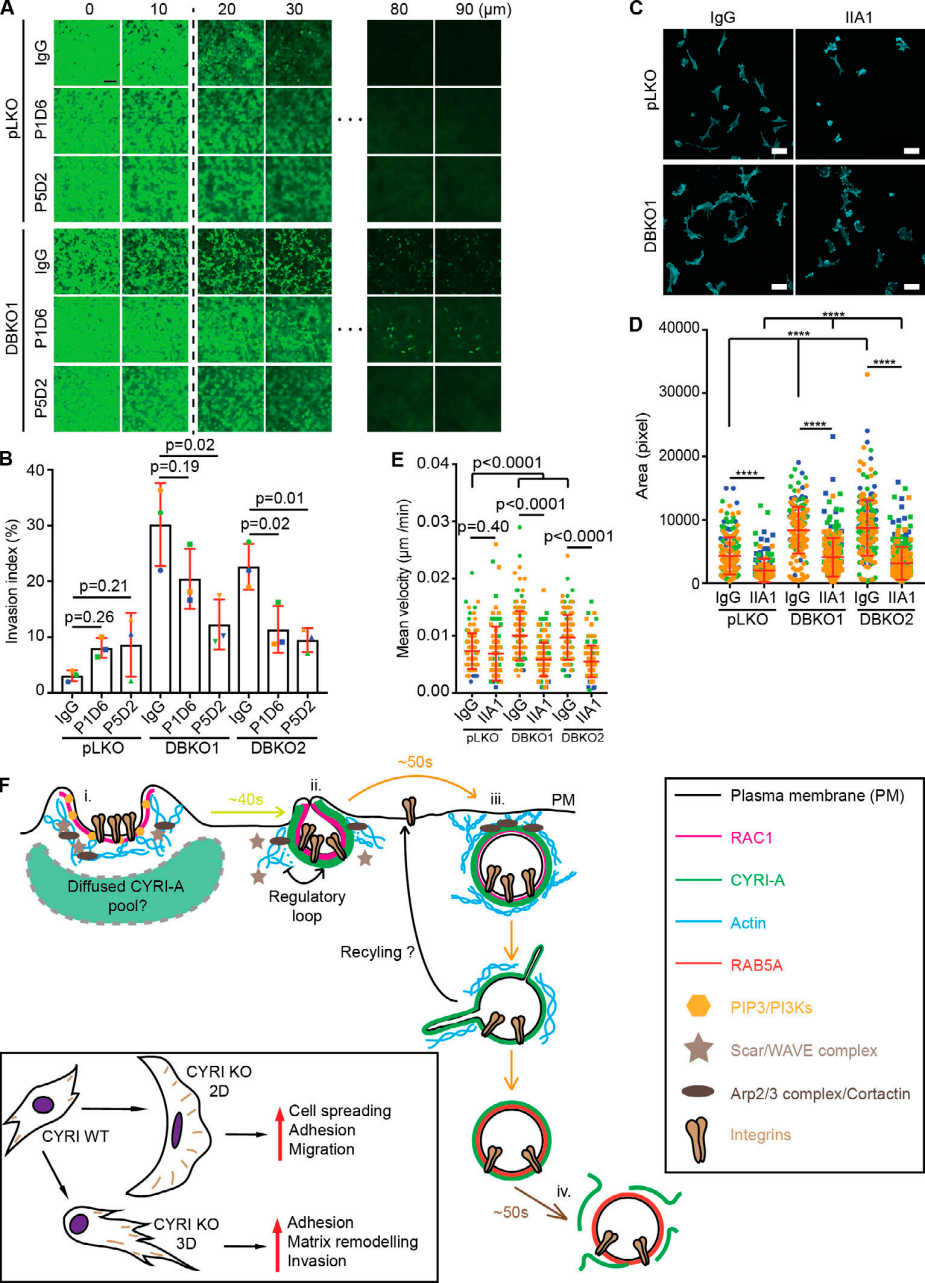
**α5β1 integrins are important for CYRI DBKO cell invasion.(A and B)** Inverted invasion assay of control pLKO and CYRI DBKO A-673 cells with CMF plug treated with IgG (negative control), P1D6 (α5-blocking antibody), or P5D2 (β1-blocking antibody; A). The depth of invasion beyond 10 µm was quantified as an invasion index shown in B. Data from three independent experiments. Each experiment is color-coded. Mean ± SD. Statistical analysis using one-way ANOVA with Tukey’s multiple comparison test. Scale bar = 100 µm. **(C and D)** Immunofluorescence images of control pLKO or CYRI DBKO A-673 cells treated with 5 µg/ml of control IgG antibody or α5β1-blocking antibody IIA1 and stained for F-actin (cyan). Cell spread area was measured and plotted in D. Data from at least 10 random fields of view per experiment in a total of three independent experiments. Each experiment is color-coded. Mean ± SD. Statistical analysis using unpaired *t* test or one-way ANOVA with Tukey’s multiple comparison test where appropriate. Scale bar = 50 µm. **(E)** Treating DBKO A-673 cells with α5β1-blocking antibody IIA1 shows the reduction in their migration ability on 2D fibronectin substrate. Data from at least 30 cells per experiment in a total of three independent experiments. Each experiment is color-coded. Statistical analysis using unpaired *t* test or one-way ANOVA with Tukey’s multiple comparison test where appropriate. Mean ± SD. ****, P < 0.0001. **(F)** The current working model: (i) RAC1, the Scar/WAVE complex, the Arp2/3 complex, and actin drive the formation of macropinocytic cups. (ii) RAC1 activity increases lead to the recruitment of CYRI-A from a diffuse pool. (iii) CYRI-A is recruited to the nascent macropinocytic cups or just after the cups have closed by active RAC1, where it dampens down RAC1 activity, terminates actin polymerization, and allows for the completion of the macropinosomes, taking surface integrins into the cytoplasm. (iv) CYRI-A slowly disappears while RAB5A starts to appear. Cancer cells lacking both CYRIs retain more integrins on their surface, which can help them adhere to the surrounding matrix to assist their migration and invasion.

Increased levels of α5 and β1 integrins have been associated with increased invasiveness and survival of multiple types of cancer (Bianchi-Smiraglia et al., 2013; Clark et al., 2005; Mierke et al., 2011; Nam et al., 2010; Paul et al., 2015). Building on these observations and our migration data (Fig. 3, D–H; and Fig. S1, F and G), we tested the invasive capacity of the CYRI DBKO A-673 cells into collagen plugs (Timpson et al., 2011). Loss of CYRI proteins significantly increased invasion by approximately threefold (10% for DBKO and 3% for controls; Fig. 10, A and B). Blocking the activity of the integrin α5β1 using the blocking antibody IIA1 dramatically reduced the invasion of the DBKO cells, but not controls, into collagen-Matrigel-fibronectin (CMF) plugs in inverted invasion assays (Fig. 10, C and D). Individually inhibiting each subunit with specific blocking antibodies also reduced invasion (Fig. S5, A and B). On a 2D surface, pretreating cells with IIA1 blocking antibody significantly reduced their ability to adhere and spread on fibronectin matrix (Fig. S5, C and D). This suggested that in CYRI DBKO cells, integrins play a crucial role in regulating shape and adhesion. Treatment with this antibody, however, only affected the migration ability of the DBKO cells, but not the control pLKO, in a 2D random migration assay (Fig. S5 E), in agreement with our invasion data (Fig. 10, C and D). Integrins also provide cancer cells with increased resistance to anoikis, a programmed cell death process triggered by the lack of adhesions, through triggering FAK activation on the endosomal membrane (Alanko et al., 2015). Culturing the control and two DBKO cell lines in the low-attachment condition of agarose showed that indeed the DBKO cells formed larger colonies (Fig. 10, E and F). Overall, our data provide a mechanism linking the function of CYRIs in macropinocytosis to the invasive capacity of cancer cells through modulating integrin trafficking and signaling (Fig. S5 F).

## Discussion

CYRI proteins specifically bind to RAC1 and oppose recruitment and activation of Scar/WAVE complex in lamellipodia (Fort et al., 2018). Fort et al. (2018) showed that CYRI-B can decrease RAC1 activation in cells by an unknown mechanism and likely sequester RAC1 away from interaction with Scar/WAVE or somehow disrupt RAC1 signaling to Scar/WAVE (Fort et al., 2018). However, the cellular functions of the related protein CYRI-A have never been specifically described, and nothing is known about the dynamics of CYRI proteins in live cells. CYRI-A, encoded by the *FAM49A* gene, has previously been linked to nonsyndromic oral clefts and craniofacial abnormalities (Chen et al., 2018; Leslie et al., 2016; Azevedo et al., 2020), suggesting an important role in developmental and perhaps morphogenetic processes, but a mechanistic understanding of its cellular function is currently missing. Here, we model CYRI-A into the recently solved structure of CYRI-B (Yelland et al., 2021; Kaplan et al., 2020) and show that the overall structure is likely similar. We show biochemically that CYRI-A interacts specifically with active RAC1 via a conserved double arginine motif, but with a higher affinity than CYRI-B. CYRI-A expression can rescue the effects of CYRI-B deletion on lamellipodia and spreading, reflecting that they likely have overlapping functions. Depletion of both CYRI-A and CYRI-B together has a stronger effect on lamellipodia and cell migration than depletion of either protein independently. We thus place CYRI-A as a paralog of CYRI-B, with broadly similar behavior, including specifically interacting with active RAC1 and opposing recruitment and activation of Scar/WAVE complex at the leading edge of cells. We also developed a new tool to study for the first time the dynamics of CYRI proteins, which revealed an unexpected dynamic role of CYRI-A in the early stages of macropinocytosis, during cup formation and internalization, with implications for integrin trafficking, cell migration, and invasion.

The interplay between signaling and cytoskeletal dynamics that drives macropinocytosis involves PI3K activation and PIP3 signaling, leading to the activation of RAC1 (likely via recruitment of a RAC1 GEF) and thus the Scar/WAVE complex. The resulting actin assembly drives the formation of membrane ruffles and macropinosomes (Veltman et al., 2016). As we previously found that CYRI-B enhances dynamics at the leading edge of the cell by opposing actin-driven membrane expansion (Fort et al., 2018), it is also possible that CYRI has a direct role in promoting membrane ruffling that leads to macropinocytic cup formation. This could imply coordinating with PI3K, which drives dorsal ruffle structures (Corallino et al., 2018), but this requires further study. We observed dynamic recruitment of CYRI-A at macropinocytic cups, persisting until after engulfment. While we were not able to distinguish whether CYRI-A is present both before and after cup closure, using markers of the various stages of macropinocytosis, we placed CYRI-A after PI3K and immediately downstream of RAC1 activation and the initiation of actin assembly at the forming macropinocytic cup. CYRI-A is recruited before and only briefly present coincidentally with RAB5A, a marker of the early endosomes. By contrast, CYRI-B appeared to be more evenly distributed at the plasma membrane and on internal tubular and vesicular structures. So, while CYRI-A and CYRI-B can compensate for each other in aspects of cell motility, they appear to be differently regulated. However, we cannot rule out that the internally inserted fluorescent protein tag affects CYRI-A and CYRI-B differently and might affect their localization or even their activity.

Both the activation and deactivation of RAC1 are required for the completion of macropinocytosis. For example, when RAC1 activation is sustained using photoactivation, the macropinocytic cups were arrested until RAC1 activation was switched off (Fujii et al., 2013). In macrophages, RAC1 GAPs are frequently paired with GEFs (Denk-Lobnig and Martin, 2019) and are needed for the completion of phagocytosis of large particles (Schlam et al., 2015), presumably to shut down RAC1 signaling and to allow actin disassembly. However, a specific inhibitor of RAC1 at the macropinocytic cup has never been discovered. Here, we show that CYRI-A rapidly accumulates on macropinocytic cups and nascent macropinosomes harboring activated RAC1, where it presumably sequesters RAC1 or disrupts its activity toward Scar/WAVE, promoting actin disassembly. Our studies suggest that CYRI-A and actin form a feedback loop, as blocking actin dynamics using Latrunculin A or Cytochalasin D decreased CYRI-A’s lifetime on the cups/vesicles, while the presence of CYRI-A decreased the lifetime of actin. Interestingly, a proteomic analysis of macropinosomes done in *Dictyostelium* also implicated CYRI (then known only as Fam49; Journet et al., 2012), suggesting that this function is likely to be conserved.

We speculate that CYRI-A opposing Scar/WAVE and actin dynamics is important for the loss of the dense actin coat around the newly formed/forming macropinosome to allow it to move inside of the cell and begin its maturation process. The actual mechanism of how CYRI proteins oppose RAC1 activation of Scar/WAVE complex still needs further study. While CYRI proteins are present in cells at sufficient concentrations to compete with Scar/WAVE complex (Fort et al., 2018), they show a relatively low affinity in vitro for RAC1, and we have not detected GTPase activating activity toward RAC1 directly (unpublished data). It would be interesting to know if CYRI-A cooperates with another recently described regulator of actin on macropinosomes, Phafin2 (Schink et al., 2017 *Preprint*). Phafin2 is described as a coincidence detecting protein important for the removal of the actin coat and, as such, may have interplay with CYRI proteins.

DBKD of CYRI-A and CYRI-B gave a robust enhancement of cell spreading and concomitant increase in surface α5β1 integrin. Integrins are internalized by multiple different routes, including macropinocytosis (Gu, et al., 2011) and the CLIC-GEEC pathway (Moreno-Layseca et al., 2020 *Preprint*), where they can be rapidly recycled back to the surface or targeted for degradation in lysosomes (Yu et al., 2012; Zech et al., 2011). It is unclear whether certain routes of uptake, such as clathrin-mediated endocytosis or macropinocytosis, might favor degradation versus recycling, but we find that by either depleting CYRIs or treating cells with PI3K inhibitor LY293002, we slow down the internalization of active integrin as detected with the SNAKA51 antibody (Fig. 9). The SNAKA51 antibody preferentially recognizes active integrin α5β1 at fibrillar adhesions that are bound to fibronectin (Clark et al., 2005). Active ligand-bound α5β1 integrin is also internalized in an Arf4-Scar/WAVE–dependent manner for trafficking to lysosomes (Rainero et al., 2015). Integrin engagement and recycling are important regulators of cancer cell invasive migration (Yu et al., 2012; Caswell et al., 2008; Dozynkiewicz et al., 2012; Paul et al., 2015). Loss of CYRI-A and -B promoted cancer cell invasion, presumably because of excess active integrins on the cell surface and perhaps higher overall levels of RAC1 activation and engagement of the Scar/WAVE complex at the cell’s leading edges. We cannot, however, entirely rule out the effects of other endocytic pathways in contributing to integrin trafficking in our KO cells. Future experiments should address in more detail the connection between CYRI-mediated endocytosis with other pathways such as clathrin-mediated endocytosis, one of the major integrin trafficking processes. Also, given the role of CYRIs in membrane dynamics, it would be interesting to study whether CYRIs could affect the mechanical properties of the cells. Given the larger spreading area and the higher level of surface integrins, one might speculate an effect of CYRI KO on membrane tension, which could also result in changes in membrane receptor trafficking. While our study implicates CYRI proteins in trafficking of integrins via macropinocytosis, we do not think it likely that integrins are the only cargo, as macropinocytosis involved uptake of multiple membrane receptors.

Overall, our study points to CYRI proteins as regulators of actin dynamics not only at the plasma membrane for lamellipodia dynamics, as previously described (Fort et al., 2018), but also as regulators of macropinocytic uptake. Our model in Fig. S5 F describes how CYRI-A is specifically recruited from a more diffuse localization to bright flashes appearing at the ruffle-to-cup and early vesicle stages of macropinocytosis. CYRI-A binds to and is recruited by its interaction with active RAC1, similar to the previously described “local inhibitor” postulated by Meinhardt (1999). Thus, the activation signal also triggers local recruitment of an inhibitor, CYRI-A, which opposes the activation of Scar/WAVE complex by RAC1 and facilitates the closing and engulfment of macropinocytic cups and the loss of the dense actin meshwork that was underpinning the ruffle protrusions. We show that CYRI proteins’ role in macropinocytosis impacts on the level of surface integrins displayed on cells and thus their ability to adhere, spread, and invade into 3D matrix. This could have important implications for cancers where RAC1 pathways are disrupted or enhanced, such as in melanomas where RAC1 activating mutations are considered to be drivers (Cannon et al., 2020).

## Materials and methods

### Cell line generation, maintenance, and growth conditions

COS-7, CHL-1, and HEK293T cells were grown in DMEM (#21969-035; Gibco) supplemented with 10% FBS (#10270-106; Gibco), 1× glutamine (#25030-032; Gibco), and 1× penicillin/streptomycin (#15140122; Life Technologies). A-673 cells were a kind gift from Dr. Susan Burchill (Leeds Institute, Leeds, UK) and were grown in the same medium but with 0.5× glutamine. Single CYRI CRISPR A-673 was generated using Lentiviral CRISPR vector (hSpCas9-2A-Puro, #62988; Addgene) and selected using 1 µg/ml puromycin (#ant-pr-1; InvivoGen) for 1 wk. Double CYRI CRISPR A-673 was generated using a combination of the hSpCas9-2A-Puro and a modified version containing a Blasticidin resistant gene (a kind gift from Dr. Stephen Tait, CRUK Beatson Institute, Glasgow, UK). DBKO cells were selected by using a combination of 1 µg/ml puromycin and 6 µg/ml Blasticidin (#ant-bl-10p; InvivoGen) for 1 wk.

For maintenance, cells were kept in the presence of all antibiotics to prevent incomplete selection. For passaging, cells were washed with PBS, and 500 µl of 0.25% trypsin (#15090046; Gibco) was added and incubated at 37°C for 2 min. Trypsin was blocked by adding 5 ml medium containing 10% serum, and cells were split in a 1:10 ratio on day 1 and 1:5 on day 5. For experimental testing, cells were grown in antibiotic-free medium. Cells were grown at 37°C in a 5% CO_2_ incubator.

For all experiments with multiple repeats, cells of different passages were considered biological replicates. Cells of different origins (HEK293T, COS-7, and CHL-1) were used to confirm the reproducibility of our observations in live-cell imaging experiments. All cells were from ATCC and were authenticated using short tandem repeat profiling. All cells were tested for mycoplasma with qPCR and were confirmed to be negative.

### sgRNA and siRNA

sgRNAs for CRISPR were designed using the Zhang laboratory website (https://zlab.bio/guide-design-resources) and were then purchased through Thermo Fisher Scientific: h49A-sgRNA2.1, 5′-CAC​CGC​CTG​AAG​GAC​GGC​GCT​GAT​C-3′; h49A-sgRNA2.3, 5′-CAC​CGC​TGC​AGG​CTT​ACA​AAG​GCG​C-3′; and h49B-sgRNA4.1, 5′-CAC​CGC​GAG​TAT​GGC​GTA​CTA​GTC​A-3′. siRNAs were purchased from Qiagen: Hs_FAM49A_5 FlexiTube siRNA (#SI03150210), 5′-ATC​GAT​ATG​AAA​GGC​TGC​ATA-3′; Hs_FAM49A_9 FlexiTube siRNA (#SI05122656), 5′-CAG​ATT​GAT​GTT​AAT​ACT​TGT-3′; Hs_FAM49B_7 FlexiTube siRNA (#SI04359369), 5′-ATA​GAA​GAA​CAT​TGA​GTC​GTA-3′; and AllStars Neg. Control siRNA (20 nmol; #0001027281).

For CRISPR gene deletion, lentivirus containing the CRISPR-Cas9 construct was generated using HEK293T cells. In brief, 1.5 × 10^6^ HEK293T cells were plated on a 10-cm Petri dish. Cells were transfected with 10 µg of CRISPR construct containing sgRNA targeting the gene of interest, 7.5 µg of pSPAX2, and 4 µg of pVSVG packaging plasmid using standard calcium precipitation protocol (#K278001; Invitrogen) and grown in 20% serum-containing medium for 24 h. Conditioned medium was filtered through a 0.45-µm filter and mixed with 2.5 µl polybrene per 6 ml of conditioned medium before being added to the targeting cells. The infection process was repeated a second time before antibiotics were added for 1 wk for selection.

For siRNA gene silencing, one reaction, 20 nmol of siRNA was mixed with 7 µl of Lullaby (#LL71000; Oz Biosciences) and incubated in serum-free medium in a total volume of 200 µl for 20 min before adding to 3 × 10^5^ cells growing in 1,800 µl of medium. The process was repeated after 48 h, and cells were split if becoming too confluent. 24 h after the second treatment, cells were used for further experiments.

### Lipofectamine plasmid transfection

For one reaction, 2 µg of DNA plasmid was mixed with 5 µl of Lipofectamine 2000 (#11668019; Thermo Fisher Scientific) in serum-free medium to a total volume of 200 µl and incubated at room temperature for 5 min before adding to 3 × 10^5^ cells in a six-well plate. Cells were incubated at 37°C, 5% CO_2_, and the transfection efficiency was assessed after at least 18 h.

### Cell fixation and immunofluorescence

Glass coverslips were treated in 70% HNO_3_ (nitric acid) for 30 min before washing with a copious amount of water. Coverslips were then coated with 1 mg/ml of fibronectin (#F1141; Sigma-Aldrich) for 2 h and washed with PBS. Cells were seeded on coverslips and left to settle for 4 h before being fixed in 4% PFA. Cells were permeabilized with buffer containing 20 mM glycine and 0.05% Triton X-100 in PBS for 5 min before being incubated with primary antibody (1:200 dilution) and then secondary antibodies (1:200 dilution), phalloidin (1:100 dilution), and DAPI (1:1,000 dilution). Cells were then mounted on a glass slide using ProLong Diamond Antifade Mountant (#P36961; Invitrogen).

### CYRI-B CRISPR COS-7 cell CYRI-A rescue experiments

CYRI-B CRISPR COS-7 cells were transfected with a control GFP-FLAG or CYRI-A-FLAG construct using Lipofectamine protocol for 24 h before cells were seeded on acid-treated fibronectin-coated coverslips for 4 h. Cells were fixed and stained using the immunofluorescent protocol described above. The average Arp2/3 signals at the cell periphery of the transfected cells was measured using the freehand selection tool with a width of 1 pixel on 8-bit images. The cell spread area was measured by manually outlining the selected cells, then the background was removed before a constant threshold was applied.

### Cell shape analysis

A-673 cells, either CRISPR/Cas9 KO or siRNA treated, were plated on fibronectin-coated coverslips for 4 h before being fixed and stained for DAPI and phalloidin as described above. Images were acquired using a Zeiss710 20× objective. At least 10 random fields of view per sample were imaged. All images were then fed into CellProfiler 3.0.0 software to measure the spreading area.

### Integrin immunofluorescent quantification

A-673 cells were seeded on fibronectin-coated coverslips overnight. The next day, the cells were fixed and stained using the immunofluorescence protocol described above. The number and size of integrin adhesion sites were extracted using the autothreshold function RenyiEntropy white in ImageJ applied on 8-bit images. The number of integrin adhesion sites was the absolute number per cell. The size was normalized to the size of the selected cell.

### Image-based integrin internalization assay

The assay was based on the internalization assay described in Pietilä et al. (2019) with a slight modification. In brief, A-673 cells were grown on fibronectin-coated coverslips overnight to allow the cells to fully adhere. The next day, cells were washed with ice-cold PBS and incubated with SNAKA51 antibody in ice-cold HBSS (8 g NaCl, 0.4 g KCl, 0.14 g CaCl_2_, 0.1 g MgSO_4_.7H_2_O, 0.1 g MgCl_2_.6H_2_O, 0.06 g Na_2_HPO_4_.2H_2_O, 0.06 g KH_2_PO_4_, 1.0 g glucose, 0.35 g NaHCO_3_, and H_2_O to 1,000 ml) for 1 h on ice and covered in aluminum foil. Internalization was induced by adding 1 ml of 37°C serum-free medium, and cells were quickly transferred to a 37°C incubator for 30 min. For the control as 0 min, the unbound antibody was washed with ice-cold PBS before the bound antibody was stripped using acid wash (0.2 M acetic acid and 0.5 M NaCl, pH 2.5, in PBS) for 1.5 min. After 30 min, cells were removed from the incubator and washed with 1 ml of ice-cold PBS to terminate any endocytosis. Uninternalized antibodies on the cells were acid-stripped, and both conditions were fixed with 4% PFA. Cells were then subjected to our standard immunofluorescence protocol as described above and stained for DAPI and the secondary antibody for integrins. For image acquisition, a z-stack of 0.18 µm per slice was taken with an AiryScan Zeiss880 using a Plan-Apochromat 63×/1.4 oil differential interference contrast (DIC) M27 objective lens. Healthy cells were selected based on the morphology of the nucleus. Acquired images were deconvoluted using Zen Black software before analysis. A maximum-intensity projection was generated using the 3D Project function from ImageJ and then converted to an 8-bit image. The pipeline for semiautomated quantification was set up. The cell outline was manually drawn using the freehand selections tool. The outside background was removed. The autothreshold function MaxEntropy in ImageJ was used to identify internalized signals. Enhanced-contrast images were used to define cell area. The level of internalization was quantified by measuring the area of internalized signal and was normalized to the area of the selected cell.

For the rescue experiment, CYRI DBKO A-673 cells were transfected with either GFP or P16-GFP-CYRI-A overnight. Cells were seeded on fibronectin-coated coverslips overnight before being subjected to the same internalization assay protocol. A z-stack of 0.64 µm per slice was taken with an AiryScan Zeiss880 using a Plan-Apochromat 63×/1.4 oil DIC M27 objective lens. The same pipeline was applied to quantify the internalization signal using either MaxEntropy or Intermodes for autothresholding.

For the inhibitor experiment, P16-GFP-CYRI-A–rescued CYRI DBKO A-673 cells were used due to the control pLKO cells were very sensitive to PI3K inhibition with LY294002. Rescued cells were not induced for internalization, were induced for internalization for 30 min using 37°C serum-free medium, or were induced for internalization in the presence of 10 µM of LY294002. The same image acquisition and image analyses were applied for quantification.

### Macropinocytosis assay

Cells were transfected with siRNA targeting both *CYRI-A* and *CYRI-B* genes for 48 and 24 h before seeding onto fibronectin-coated coverslips overnight. The next day, cells were washed three times with ice-cold PBS before being incubated with 0.2 mg/ml dextran 70 kD 488 (#D1822; Thermo Fisher Scientific) for 15 or 30 min in serum-free medium at 37°C. Cells were then washed five times in ice-cold PBS and fixed for 15 min using 4% PFA before staining with DAPI for 30 min. 10 random fields of view were taken using a Zeiss710 40× objective and analyzed using a semiautomated ImageJ macro. The outline of the cell clusters was determined by increasing the brightness with ImageJ and outlining using the freehand selection tool. The background was removed. The autothreshold function MaxEntropy in ImageJ was used to measure the internalized dextran signal. The macropinocytic index was calculated by normalizing the internalized dextran signal to the total area of the cell cluster for each field of view (Commisso et al., 2014).

For overexpression experiments in Figs. 8 and 9, scramble and DBKD COS-7 cells were transfected with either P16-mCherry-CYRI-A WT or RRDD mutant and compared with nontransfected scrambled and DBKD cells. Images were taken using a Zeiss710 63× objective. Quantification was done as described above.

### Western blotting

Cells were grown to 80% confluence in a six-well plate and lysed with radioimmunoprecipitation assay buffer (150 mM NaCl, 10 mM Tris-HCl, pH 7.5, 1 mM EDTA, 1% Triton X-100, and 0.1% SDS) supplemented with protease and phosphatase inhibitor cocktails (#78438, #78427; Thermo Fisher Scientific). Lysates were spun at maximum speed for 10 min before protein levels were measured using Precision Red (#ADV02; Cytoskeleton). Samples were run using precast 4–12% NuPAGE Bis-Tris Acrylamide Gels (#NP0321; Thermo Fisher Scientific) and transferred to a 0.45-µm nitrocellulose membrane (#10600002; GE Healthcare). Membranes were blocked in 5% BSA in TBS + 0.1% Tween-20 for 30 min before being incubated with specific primary antibodies overnight and the corresponding fluorescently conjugated secondary antibody for 1 h (#A21206 and #A10038, Invitrogen; #SA5-35521 and #SA5-35571, Thermo Fisher Scientific) before being analyzed using Image Studio Lite (LiCor).

### GST-Trap pulldown

For small-scale GST-tagged protein purification, BL21 competent *Escherichia coli* was grown until OD 0.4 and induced with 200 µM IPTG overnight at room temperature. Cells were pelleted and lysed using sonication in buffer containing 50 mM Tris, pH 7.5, 50 mM NaCl, 5 mM MgCl_2_, and 250 µM DTT. The lysates were collected by ultracentrifugation at 20,000 rpm for 20 min. The lysates were added directly on glutathione sepharose 4B beads (#17075601; GE Healthcare) and incubated for 2 h at 4°C. If GST-tagged proteins needed to be eluted from the beads, elution buffer containing 100 mM NaCl, 25 mM Tris, pH 8, 5 mM MgCl_2_, 10 mM Glutathione, and 0.1% Triton X-100 was added and incubated overnight. At least 6 × 10^6^ COS-7 cells on a 15-cm Petri dish were transfected with GFP-tagged constructs overnight using Lipofectamine 2000 as described above and lysed the next day using lysis buffer containing 100 mM NaCl, 25 mM Tris-HCl, pH 7.5, and 5 mM MgCl_2_ supplemented with 0.5% NP-40 and protease and phosphatase inhibitors. Lysates were cleared by centrifugation, and 1.5 mg of protein lysate was incubated with GST beads for 2 h before being subjected to Western blotting as described. The signal of the pulldown was normalized to the GFP signal.

### Recombinant protein purification and SPR

In brief, BL21-competent *E. coli* containing MBP- or GST-tagged protein were grown to OD 0.4 and induced overnight with 200 µM IPTG at room temperature. The next day, cells were concentrated and lysed using the microfluidizer machine in lysis buffer (20 mM Tris, pH 8.0, 300 mM NaCl, 5 mM MgCl_2_, and 2 mM β-mercaptoethanol) supplemented with protease inhibitors. Cell lysates were cleared using ultracentrifugation at 20,000 rpm for 45 min. The lysates were passed through an affinity column (either MBPTrap HP, #GE28-9187-79, or GSTrap 4B, #GE28-4017-47, Sigma-Aldrich; buffer contained 20 mM Tris, 150 mM NaCl, 5 mM MgCl_2_, and 2 mM DTT) before being eluted with either 10 mM maltose or glutathione. The eluted fractions were then subjected to size-exclusion chromatography using an HiLoad16/600 Superdrex 200 pg. GST tags were digested overnight with thrombin and separated using affinity chromatography.

For SPR, MBP-tagged proteins were immobilized on Sensor chip CM5 (#BR100012, GE Healthcare), and purified untagged RAC1 Q61L was titrated at different concentrations. The response signals were recorded and fitted into a 1:1 binding model. The *K_d_* was estimated from the fitted curve.

### MBP-Trap pulldown assay

MBP-tagged proteins were purified from BL21 bacteria and conjugated to MBP-Trap agarose beads (#mbta-20; ChromoTek) similar to GST-tagged protein. 30 µl of beads were incubated with 30 µg of purified GST-tagged RAC1 in a total of 500 µl volume at 4°C for 90 min. The beads were washed five times with wash buffer before being subjected to Western blotting as described earlier. The signal of the pulldown protein was normalized to the MBP signal.

### 2D and 3D CDM migration assay

For the 2D random migration assay, six-well plates were coated with 1 mg/ml of fibronectin in PBS for 2 h before 20,000 cells were seeded and left to settle for 4 h at 37°C. Cells were imaged using a Nikon TE2000 microscope equipped with a PlanFluor 10×/0.30 objective every 15 min for 20 h. Individual cells were tracked manually over time using the MTrack ImageJ plug-in, and the velocity was calculated using Chemotaxis tool ImageJ plug-in. For IIA1 treatment, cells were incubated with 5 µg/ml of antibody (either IgG control or IIA1) in 1 ml of serum-free medium with rotation at room temperature for 1 h. Cells were then seeded on the fibronectin-coated surface for 4 h before being imaged with the same Nikon system.

For the 3D CDM migration assay, CDM was generated using telomerase-immortalized fetal foreskin (TIFF) cells. In brief, TIFF cells were seeded on a 0.2% cross-linked gelatin-coated six-well plate and fed with ascorbic acid every 2 d. TIFF cells were extracted using lysis buffer containing 20 mM NH_4_OH and 0.5% Triton X-100 in PBS with Ca^2+^ and Mg^2+^ for 2 min and DNase for 30 min. The plates were kept in PBS at 4°C until use (Cukierman et al., 2001). The same seeding, imaging, and analysis protocol was used.

### Wound-healing assay

A 96-well plate (#353072; Falcon) was coated with 1 mg/ml fibronectin for 2 h. Cells were seeded to form a confluent monolayer (70,000 cells for A-673) and left to settle for 4 h. The wound was made using the Incucyte wound maker. Cells were stained with IncuCyte Nuclight Rapid Red reagent (#4717) for total cells and IncuCyte Sytox Green reagent (#4633) for dead cells. Cells were imaged every hour for 48 h using the IncuCyte system. Analysis of wound closure was done with Zoom software (IncuCyte Biosciences, Sartorius), where the difference in the wound area from time 0 h to the final frame was calculated and then divided by the length of time to get the wound closing rate in micrometers per hour.

### Proliferation assay

10^4^ cells were seeded per well in a fibronectin-coated 96-well plate (#353072; Falcon). Cells were stained with IncuCyte Nuclight Rapid Red reagent (#4717) for total cells and IncuCyte Sytox Green reagent (#4633) for dead cells and imaged for 28 h. To calculate the growth curve, the total number of live cells was calculated by subtracting the number of Sytox-positive cells from the number of Nuclight Red–positive cells. The result was then normalized to the number of live cells from time 0. The slope was calculated using a nonlinear regression model in Prism7 software.

### xCELLigence spreading assay

xCELLigence E-plate 16 (#05469830001; Acea) was coated with 1 mg/ml of fibronectin for 1 h. Cells were trypsinized as described earlier and counted, and 10,000 cells were seeded on the plate. Immediately, the plate was moved to an Acea RTCA DP xCELLigence device, and cell spreading was measured based on electrical impedance every 5 min for 8 h.

### Live-cell superresolution fluorescence microscopy

For all live imaging experiments, cells were seeded on a 1 mg/ml fibronectin-coated 3.5-cm dish for 4 h before imaging. During the imaging process, cells in normal growth medium were kept at 37°C in a 5% CO_2_ chamber. Imaging was performed with a Zeiss 880 Laser Scanning Microscope with Airyscan with a Plan-Apochromat 63×/1.4 oil DIC M27 objective lens and deconvoluted using Zen Black Zeiss880 deconvolution software (ZEN 2.3 SP1 FP3 v14.0.17.29).

For visualization of CYRI-A and CYRI-B, cells were imaged every 9 s for 50 frames. The lifetime of CYRI-A on the cups/vesicles was calculated based on the number of frames in which the CYRI-A signal was present. For measuring the size of CYRI-positive structures, the straight-line tool from ImageJ was used. The largest diameter or the longest length was considered the size of the cups/vesicles and tubules, respectively.

For measuring the lifetime of actin, the number of frames in which the actin signal was present was counted. As many structures as could be seen were included in the analysis.

For the actin depolymerization experiment, at least 5 cells were imaged before Latrunculin A (#L5163; Sigma-Aldrich) or Cytochalasin D (#C2618; Sigma-Aldrich) was added at 1 µM. Then, at least five random cells were imaged after at least 5 min of drug treatment. Images were taken every 9 s. Cup/vesicle lifetime was calculated based on the number of frames in which the structures were present.

For the PI3K inhibition experiment, nine random cells were imaged every 30 s for 20 frames before LY294002 was added at 20 µM, and the imaging process continued for another 20 frames. The number of cups/vesicles was manually counted in all frames for every cell. For the live-cell dextran incorporation assay, cells were first imaged every 9 s before dextran (Texas Red 70,000 MW Lysine Fixable, #D1864; Invitrogen) was added at 50 µg/ml mid-imaging.

For all line graphs representing the signal intensity over time, the signal of each channel was manually measured using the freehand selection tool. The signals were then normalized to the highest value and plotted over time.

To calculate the percentage of colocalization events, the number of cups/vesicles that colocalized or did not colocalize between two signals was counted: % colocalization events = (# colocalizing events/# noncolocalizing events) × 100.

### Flow cytometry analysis

10 × 10^6^ cells were grown in a 15-cm Petri dish overnight. The next day, cells were trypsinized, washed in PBS, and filtered through a 40-µm filter. All conditions were stained with Zombie Red reagent (#423110) before being fixed in 4% PFA for 15 min. Cells were blocked in PBS supplemented with 1% serum for 30 min and then incubated with the corresponding primary antibody for 15 min at room temperature. If the primary antibodies were not conjugated with Alexa Fluor 488, cells were incubated with the secondary antibody for 15 min at room temperature before analyzing using the Attune NxT system (Thermo Fisher Scientific). For negative control, cells were incubated with anti-human mouse IgG (#400132; BioLegend). For all conditions, dual colors of the far-red and Alexa Fluor 488 flow cytometry were used to minimize spectrum overlapping. Data were imported and analyzed using FlowJo software (v. 10.6.1, BD).

### qPCR

Cells were grown to 80% confluence in a six-well plate before RNA was extracted using a standard RNA extraction protocol according to the manufacturer (RNAeasy Mini Kit, #74104; Qiagen). cDNA synthesis was performed by mixing 1 µg of RNA with qScript cDNA Synthesis Kit (#95047-100; Quantabio) and reaction conditions as follows: 5 min at 25°C, 30 min at 42°C, and 5 min at 85°C using a thermocycler. qPCR was performed with the DyNAmo HS SYBR Green qPCR kit (#F410L; Thermo Fisher Scientific) according to the manufacturer’s protocol and was done using the QuantStudio Real-Time PCR system. The ΔΔCt method was used to quantify qPCR data. All primers were purchased from Qiagen website: Hs_ITGA5_1_SG QuantiTect Primer Assay (#QT00080871), Hs_ITGB1_1_SG QuantiTect Primer Assay (#QT00068124), and Hs_ACT_2_SG QuantiTect Primer Assay (#QT01153551).

### Organotypic assay

The organotypic assay was performed based on Timpson et al. (2011). In brief, collagen was extracted from rat tails and kept in 0.5 mM acetic acid buffer at 2 mg/ml concentration. Collagen plugs were made by mixing TIFF cells with 25 ml of collagen, and the pH was adjusted to 7.2 using NaOH. Collagen plugs were allowed to contract for 8 d before being transferred to a 24-well plate. A-673 cells were prepared as usual without any trypsin present in the final solution, and 3 × 10^4^ were seeded directly on top of each plug. Medium was changed every 2 d for 5 d before the plugs were transferred onto a metal grid. A liquid–air interface was set up, with the medium acting as a chemoattractant for invasion. Medium was changed every 2 d for 14 d before the plugs were collected and processed for hematoxylin and eosin histology staining. Images were quantified using HALO software (PerkinElmer). The invasion index was quantified by measuring the invading area and normalizing it to the size of the entire tissue section.

### Inverted invasion assay

The plug was made by mixing Collagen I with Matrigel (#354234; BD) and fibronectin (CMF) in ice-cold PBS to a final concentration of 4, 4, and 1 mg/ml, respectively. 100 µl of the mixture was added to each Transwell (TKT-525-110X, C3422; Thermo Fisher Scientific) with 8-µm pore size and left to solidify at 37°C. 5 × 10^4^ A-673 cells were seeded on the underside of the Transwell and left to adhere for 4 h at 37°C. The Transwell was inverted and dipped into 500 µl of 10% serum-containing medium. The top chamber was filled with 100 µl of serum-free medium. Inhibiting antibodies were added to the top chamber at a concentration of 5 µg/ml. The cells were left to invade for 5 d before staining with 4 µM Calcein AM (#C1430; Invitrogen). Z-stack images were captured every 10 µm using the Olympus FV1000 confocal microscope with a UplanSApo 20×/0.74 objective. The invasion index was calculated by measuring the area of invading cells beyond 10 µm in the plug and then normalizing to the total area of invading cells. The area measurement was performed using a predefined threshold function in ImageJ.

### Low-attachment assay

Agarose was dissolved in PBS at 4% and autoclaved. Before the experiments, agarose was melted in the microwave and kept in a water bath at 65°C. To make the bottom layer, agarose solution was mixed with 10% serum-containing medium to the final concentration of 0.7% agarose. 1.5 ml of this was laid to make the bottom layer. While this layer was solidifying, cells were trypsinized and made into a single-cell suspension. For the top layer, for each well, 3 × 10^4^ cells were mixed with the agarose and serum-containing medium to a final agarose concentration of 0.35%. 1.5 ml of this cell suspension was quickly dispensed on top. Finally, 2.5 ml of 10% serum-containing medium was pipetted on top. The cells were grown for 10 d, with the medium changed every 2 d. Five random fields of cell spheroids per condition were imaged with a standard bright-field microscope. Image quantification was done manually by outlining the spheroids with the freehand selection tool in ImageJ, and the areas of the spheroids were calculated. Only in-focus spheroids were measured.

### Cloning and primers

To make the internal GFP or mCherry CYRI-A construct, mouse CYRI-A cDNA was cloned into the pcDNA3.1(+) vector (a kind gift from Dr. Douglas Strathdee, Beatson Institute, Glaskow, UK) using standard cloning techniques. HindIII and BamHI restriction sites were inserted just after the codon coding for proline 16 using Q5-insertion mutagenesis (#E0554; NEB) according to the protocol from the manufacturer. GFP or mCherry was amplified by PCR from the pEGFP-C1 or pmCherry-N1 vector before being inserted internally in CYRI-A.

All primers were ordered from Thermo Fisher Scientific: PCR CYRI-A, 5′-TCA​ACG​GCT​AGC​ATG​GGA​AAC​TTG​CTC​AAA​GTC-3′, 5′-TCA​ACG​CTC​GAG​TTA​CTG​AAG​CAT​CGC​TCG​AAT​CTG-3′; insert restriction sites, 5′-AAG​CTT​GCG​GGA​TCC​CAC​TTC​TTC​CTG​GAT​TTT​GAA​AAT​G-3′, 5′-GGA​TCC​CGC​AAG​CTT​TGG​ATA​ATT​CTC​AAT​TTC​CC-3′; PCR GFP or mCherry, 5′-TCA​ACG​AAG​CTT​GTG​AGC​AAG​GGC​GAG​GAG​CTG-3′, 5′-TCA​ACG​GGA​TCC​CTT​GTA​CAG​CTC​GTC​CAT​GCC-3′, 5′-TCA​ACG​AAG​CTT​GTG​AGC​AAG​GGC​GAG​GAG​G-3′; and insert Kozak sequence, 5′-GCC​ACC​ATG​GGA​AAC​TTG​CTC​AAA​GTC​C-3′, 5′-GCT​AGC​CAG​CTT​GGG​TCT-3′. The same strategy was used to clone for P17-GFP-CYRI-B, but the restriction sites were XHoI and NheI just after the codon code for proline 17: 5′-CTC​GAG​GCG​GCT​AGC​AAT​TTT​TTC​CTT​GAT​TTT​GA-3′, 5′-GCT​AGC​CGC​CTC​GAG​TGG​CCC​CTG​CTC​AAG​GTC​TG-3′. Primers for GFP-CYRI-A (1–319) and GFP-CYRI-A (29–319), 5′-TCA​ACG​CTC​GAG​TGA​TGG​GAA​ACT​TGC​TCA​AAG​TC-3′, 5′-CAT​GCA​GGA​TCC​TTA​CTG​AAG​CAT​CGC​TCG​AAT​CTG-3′, 5′-TCA​ACG​CTC​GAG​TGG​AAG​GAG​AGC​GAG​AGA​TAT​GG-3′, and 5′-CAT​GCA​GGA​TCC​TTA​TCG​AAT​CTG​TTT​GGA​AGT​CGA-3′. Primers for the mVenus-bi-cistronic vector of CYRI-A, 5′-TCA​ACG​GCT​AGC​GCC​ACC​ATG​GGA​AAC​TTG​CTC​AAA​GTC-3′, and 5′-TCA​ACG​GGA​TCC​TTA​CTG​AAG​CAT​CGC​TCG​AAT​CTG-3′. Primers for CYRI-A-FLAG, 5′-TCA​ACG​GGA​TCC​GCC​ACC​ATG​GGA​AAC​TTG​CTC​AAA​GTC-3′ and 5′-TCA​ACG​CTC​GAG​TTA​CTT​GTC​GTC​ATC​GTC​TTT​GTA​GTC​CTG​AAG​CAT​CGC​TCG​AAT​CTG-3′. Primers for MBP-CYRI-A, 5′-CGT​ATT​GGA​TCC​ATG​GGA​AAC​TTG​CTC​AAA​GTC-3′ and 5′-CGT​ATT​GAA​GCT​TCT​ACT​GAA​GCA​TCG​CTC​GAA​T-3′. GFP-PH-Grp1 was a kind gift from Dr. David Bryant (Beatson Institute, Glasgow, UK).

### Addgene plasmids

mApple-α-5-Integrin-12 (plasmid #54864; RRID:Addgene_54864; Addgene), mCherry-Clathrin LC-15 (plasmid #55019; RRID:Addgene_55019; Addgene), and mCherry-Rab5a-7 (plasmid #55126; RRID:Addgene_55126; Addgene) were gifts from Michael Davidson (Rizzo et al., 2009); pcDNA3/hArf1(WT)-mCherry (plasmid #79419; RRID:Addgene_79419; Addgene) was a gift from Kazuhisa Nakayama (Makyio et al., 2012); CAV1-mCherry (plasmid #27705; RRID:Addgene_27705; Addgene) was a gift from Ari Helenius (Hayer et al., 2010); and PH-Btk-GFP (plasmid #51463; RRID:Addgene_51463; Addgene) was a gift from Tamas Balla (Várnai and Balla, 1998).

### Antibodies

Anti–CYRI-A antibody (rabbit, Sigma-Aldrich, not in production; Western blotting), anti–CYRI-B antibody (rabbit, #HPA009076, RRID:AB_1848402; Sigma-Aldrich; Western blotting), anti-GAPDH (mouse, #MAB374, RRID:AB_2107445; Millipore; Western blotting), anti-Tubulin DM1A (mouse, #T6199, RRID:AB_477583; Sigma-Aldrich; Western blotting), anti-GST (rabbit, #2622, RRID:AB_331670; CST; Western blotting), anti-GFP (mouse, #2955, RRID:AB_1196614; CST; Western blotting), Phalloidin 488 (#A12379; Molecular Probes), anti-Integrin β 1 antibody [12G10] (rabbit, #ab30394, RRID:AB_775726; Abcam; immunofluorescent and Western blotting), recombinant anti-Integrin α 5 antibody [EPR7854] (rabbit, #ab150361, RRID:AB_2631309; Abcam; immunofluorescent and Western blotting), anti-Integrin α5 (preservative free) antibody, clone SNAKA51 (mouse, #MABT201; Millipore; immunofluorescent, internalization assay, and flow cytometry), Alexa Fluor 488 anti-human CD29 antibody TS2/16 (mouse, #303015, RRID:AB_493026; BioLegend; flow cytometry), Alexa Fluor 488 anti-mouse CD49e Antibody (rat, #103810, RRID:AB_528839; BioLegend; flow cytometry), anti-MMP-14 antibody, catalytic domain, clone LEM-2/63.1 (mouse, #MAB3329, RRID:AB_570600; Millipore; flow cytometry), donkey anti-rabbit 680-nm (#A10043, AB_2534018; Invitrogen; Western blotting), donkey anti-mouse 680-nm (#A10038, AB_2534014; Invitrogen; Western blotting), goat anti-mouse 800-nm (#SA5-35521, AB_2556774; Thermo Fisher Scientific; Western blotting), and goat anti-rabbit 800-nm (#SA5-35571, AB_2556775; Thermo Fisher Scientific; Western blotting).

### Statistical analysis

All statistical analyses were performed with Prism 7. All data points were pooled from independent experiments or cells, and then the appropriate statistical test was performed. Each experiment is color coded. Normality was assessed by inspecting the shape of the distribution. Parametric tests were used because they are less affected by deviation from normal distribution and are more robust than nonparametric tests (McDonald, 2014).

### Online supplemental material

Fig. S1 shows that untagged CYRI-A could rescue CYRI-B KO phenotype, that siRNA KD of CYRIs gave similar results as CRISPR, and the tagging strategy of our internal CYRI construct. Fig. S2 shows the localization of CYRI-B, the similar relation between CYRI-A and RAB5A in HEK293T cells, that CYRI-A positive cups/vesicles contain dextran, and that these cups/vesicles were of similar size to cells expressing only GFP. Fig. S3 shows that both CYRI-A and CYRI-B cups/vesicles were distinct from other canonical endocytic pathways and that CYRI-A recruitment was preceded by PIP3 production and membrane invagination. Fig. S4 shows the increase in surface expression of integrin α5 and β1 in CYRI DBKO cells, that non-RAC1 binding mutant CYRI-A could not rescue the spreading phenotype, and that integrins were localized to CYRI-positive cups/vesicles. Fig. S5 shows the working model and that the invasion of CYRI DBKO cells could be inhibited by integrin blocking antibodies. Video 1 shows the localization of CYRI-A in cells, its RAB5A preceding recruitment, and the macropinocytic nature of these cups/vesicles. Video 2 shows the the localization of CYRI-A to macropinocytic structures prior to RAB5A recruitment. Video 3 shows the CYRI-A–positive structures are distinct from clathrin-, caveolin-, and ARF1-mediated endocytosis. Video 4 shows CYRI-A regulates actin dynamics at the macropinocytic structures. Video 5 shows the knockdown of CYRI proteins affects actin dynamics at macropinocytic structures. Video 6 shows the effects of actin polymerization inhibition on CYRI-A recruitment and lifetime. Video 7 shows the relation between CYRI-A and RAC1 and how its recruitment to macropinocytic cups/vesicles depends on its ability to bind active RAC1. Video 8 shows the relation between CYRI-A and PIP3 and how its recruitment to macropinocytic cups/vesicles depends on PI3K activity. Video 9 shows the localization of integrins on CYRI-positive cups/vesicles.

## References

[bib1] Alanko, J., A. Mai, G. Jacquemet, K. Schauer, R. Kaukonen, M. Saari, B. Goud, and J. Ivaska. 2015. Integrin endosomal signalling suppresses anoikis. Nat. Cell Biol. 17:1412–1421. 10.1038/ncb3250 26436690 PMC4890650

[bib2] Araki, N., Y. Egami, Y. Watanabe, and T. Hatae. 2007. Phosphoinositide metabolism during membrane ruffling and macropinosome formation in EGF-stimulated A431 cells. Exp. Cell Res. 313:1496–1507. 10.1016/j.yexcr.2007.02.012 17368443

[bib3] Araki, N., M.T. Johnson, and J.A. Swanson. 1996. A role for phosphoinositide 3-kinase in the completion of macropinocytosis and phagocytosis by macrophages. J. Cell Biol. 135:1249–1260. 10.1083/jcb.135.5.1249 8947549 PMC2121091

[bib4] Azevedo, C.M.S., R.A. Machado, H. Martelli-Júnior, S.R.A. Reis, D.C. Persuhn, R.D. Coletta, and A.L.C.A. Rangel. 2020. Exploring GRHL3 polymorphisms and SNP-SNP interactions in the risk of non-syndromic oral clefts in the Brazilian population. Oral Dis. 26:145–151. 10.1111/odi.13204 31564061

[bib5] Bianchi-Smiraglia, A., S. Paesante, and A.V. Bakin. 2013. Integrin β5 contributes to the tumorigenic potential of breast cancer cells through the Src-FAK and MEK-ERK signaling pathways. Oncogene. 32:3049–3058. 10.1038/onc.2012.320 22824793 PMC3481019

[bib6] Bloomfield, G., and R.R. Kay. 2016. Uses and abuses of macropinocytosis. J. Cell Sci. 129:2697–2705. 27352861 10.1242/jcs.176149

[bib7] Bohdanowicz, M., and S. Grinstein. 2013. Role of phospholipids in endocytosis, phagocytosis, and macropinocytosis. Physiol. Rev. 93:69–106. 10.1152/physrev.00002.2012 23303906

[bib8] Buckley, C.M., and J.S. King. 2017. Drinking problems: mechanisms of macropinosome formation and maturation. FEBS J. 284:3778–3790. 10.1111/febs.14115 28544479

[bib9] Campa, C.C., E. Ciraolo, A. Ghigo, G. Germena, and E. Hirsch. 2015. Crossroads of PI3K and Rac pathways. Small GTPases. 6:71–80. 10.4161/21541248.2014.989789 25942647 PMC4601376

[bib10] Cannon, A.C., C. Uribe-Alvarez, and J. Chernoff. 2020. RAC1 as a Therapeutic Target in Malignant Melanoma. Trends Cancer. 6:478–488. 10.1016/j.trecan.2020.02.021 32460002 PMC7380552

[bib11] Canton, J. 2018. Macropinocytosis: New Insights Into Its Underappreciated Role in Innate Immune Cell Surveillance. Front. Immunol. 9:2286. 10.3389/fimmu.2018.02286 30333835 PMC6176211

[bib12] Carlsson, A.E. 2018. Membrane bending by actin polymerization. Curr. Opin. Cell Biol. 50:1–7. 10.1016/j.ceb.2017.11.007 29207306 PMC5911415

[bib13] Caswell, P.T., M. Chan, A.J. Lindsay, M.W. McCaffrey, D. Boettiger, and J.C. Norman. 2008. Rab-coupling protein coordinates recycling of alpha5beta1 integrin and EGFR1 to promote cell migration in 3D microenvironments. J. Cell Biol. 183:143–155. 10.1083/jcb.200804140 18838556 PMC2557049

[bib14] Caswell, P.T., S. Vadrevu, and J.C. Norman. 2009. Integrins: masters and slaves of endocytic transport. Nat. Rev. Mol. Cell Biol. 10:843–853. 10.1038/nrm2799 19904298

[bib15] Chattaragada, M.S., C. Riganti, M. Sassoe, M. Principe, M.M. Santamorena, C. Roux, C. Curcio, A. Evangelista, P. Allavena, R. Salvia, . 2018. FAM49B, a novel regulator of mitochondrial function and integrity that suppresses tumor metastasis. Oncogene. 37:697–709. 10.1038/onc.2017.358 29059164 PMC5808099

[bib16] Chen, C., Q. Guo, J. Shi, X. Jiao, K. Lv, X. Liu, Y. Jiang, X. Hui, and T. Song. 2018. Genetic variants of MGMT, RHPN2, and FAM49A contributed to susceptibility of nonsyndromic orofacial clefts in a Chinese population. J. Oral Pathol. Med. 47:796–801. 10.1111/jop.12758 29949196

[bib17] Chertkova, A.O., M. Mastop, M. Postma, N. Van Bommel, S. Van Der Niet, K.L. Batenburg, L. Joosen, T.W.J. Gadella, Y. Okada, and J. Goedhart. 2020. Robust and Bright Genetically Encoded Fluorescent Markers for Highlighting Structures and Compartments in Mammalian Cells. BioRxiv. (Preprint posted January 13, 2020.) 10.1101/160374

[bib18] Clark, K., R. Pankov, M.A. Travis, J.A. Askari, A.P. Mould, S.E. Craig, P. Newham, K.M. Yamada, and M.J. Humphries. 2005. A specific alpha5beta1-integrin conformation promotes directional integrin translocation and fibronectin matrix formation. J. Cell Sci. 118:291–300. 10.1242/jcs.01623 15615773 PMC3329624

[bib19] Commisso, C., R.J. Flinn, and D. Bar-Sagi. 2014. Determining the macropinocytic index of cells through a quantitative image-based assay. Nat. Protoc. 9:182–192. 10.1038/nprot.2014.004 24385148 PMC4103788

[bib20] Condon, N.D., J.M. Heddleston, T.L. Chew, L. Luo, P.S. McPherson, M.S. Ioannou, L. Hodgson, J.L. Stow, and A.A. Wall. 2018. Macropinosome formation by tent pole ruffling in macrophages. J. Cell Biol. 217:3873–3885. 10.1083/jcb.201804137 30150290 PMC6219714

[bib21] Cooper, J., and F.G. Giancotti. 2019. Integrin Signaling in Cancer: Mechanotransduction, Stemness, Epithelial Plasticity, and Therapeutic Resistance. Cancer Cell. 35:347–367. 10.1016/j.ccell.2019.01.007 30889378 PMC6684107

[bib22] Corallino, S., C. Malinverno, B. Neumann, C. Tischer, A. Palamidessi, E. Frittoli, M. Panagiotakopoulou, A. Disanza, G. Malet-Engra, P. Nastaly, . 2018. A RAB35-p85/PI3K axis controls oscillatory apical protrusions required for efficient chemotactic migration. Nat. Commun. 9:1475. 10.1038/s41467-018-03571-8 29662076 PMC5902610

[bib23] Cukierman, E., R. Pankov, D.R. Stevens, and K.M. Yamada. 2001. Taking cell-matrix adhesions to the third dimension. Science. 294:1708–1712. 10.1126/science.1064829 11721053

[bib71] De Franceschi, N., E. Peuhu, M. Parsons, S. Rissanen, I. Vattulainen, M. Salmi, J. Ivaska, and J. Pouwels. 2015. Mutually Exclusive Roles of SHARPIN in Integrin Inactivation and NF-κB Signaling. PLoS ONE. 10(11):e0143423. 10.1371/journal.pone.0143423 26600301 PMC4658161

[bib24] Denk-Lobnig, M., and A.C. Martin. 2019. Modular regulation of Rho family GTPases in development. Small GTPases. 10:122–129. 10.1080/21541248.2017.1294234 28304230 PMC6380289

[bib25] Dozynkiewicz, M.A., N.B. Jamieson, I. Macpherson, J. Grindlay, P.V. van den Berghe, A. von Thun, J.P. Morton, C. Gourley, P. Timpson, C. Nixon, . 2012. Rab25 and CLIC3 collaborate to promote integrin recycling from late endosomes/lysosomes and drive cancer progression. Dev. Cell. 22:131–145. 10.1016/j.devcel.2011.11.008 22197222 PMC3507630

[bib26] Egami, Y., T. Taguchi, M. Maekawa, H. Arai, and N. Araki. 2014. Small GTPases and phosphoinositides in the regulatory mechanisms of macropinosome formation and maturation. Front. Physiol. 5:374. 10.3389/fphys.2014.00374 25324782 PMC4179697

[bib27] Ferreira, A.P.A., and E. Boucrot. 2018. Mechanisms of Carrier Formation during Clathrin-Independent Endocytosis. Trends Cell Biol. 28:188–200. 10.1016/j.tcb.2017.11.004 29241687

[bib28] Fort, L., J.M. Batista, P.A. Thomason, H.J. Spence, J.A. Whitelaw, L. Tweedy, J. Greaves, K.J. Martin, K.I. Anderson, P. Brown, . 2018. Fam49/CYRI interacts with Rac1 and locally suppresses protrusions. Nat. Cell Biol. 20:1159–1171. 10.1038/s41556-018-0198-9 30250061 PMC6863750

[bib29] Fujii, M., K. Kawai, Y. Egami, and N. Araki. 2013. Dissecting the roles of Rac1 activation and deactivation in macropinocytosis using microscopic photo-manipulation. Sci. Rep. 3:2385. 10.1038/srep02385 23924974 PMC3737501

[bib30] Gu, Z., E.H. Noss, V.W. Hsu, and M.B. Brenner. 2011. Integrins traffic rapidly via circular dorsal ruffles and macropinocytosis during stimulated cell migration. J. Cell Biol. 193:61–70. 10.1083/jcb.201007003 21464228 PMC3082178

[bib31] Hayer, A., M. Stoeber, C. Bissig, and A. Helenius. 2010. Biogenesis of caveolae: stepwise assembly of large caveolin and cavin complexes. Traffic. 11:361–382. 10.1111/j.1600-0854.2009.01023.x 20070607

[bib66] Hinze, C., and E. Boucrot. 2018. Endocytosis in proliferating, quiescent and terminally differentiated cells. J. Cell Sci. 131(23). 10.1242/jcs.216804 30504135

[bib32] Humphreys, D., V. Singh, and V. Koronakis. 2016. Inhibition of WAVE Regulatory Complex Activation by a Bacterial Virulence Effector Counteracts Pathogen Phagocytosis. Cell Rep. 17:697–707. 10.1016/j.celrep.2016.09.039 27732847 PMC5081413

[bib33] Journet, A., G. Klein, S. Brugière, Y. Vandenbrouck, A. Chapel, S. Kieffer, C. Bruley, C. Masselon, and L. Aubry. 2012. Investigating the macropinocytic proteome of Dictyostelium amoebae by high-resolution mass spectrometry. Proteomics. 12:241–245. 10.1002/pmic.201100313 22120990

[bib34] Kaplan, E., R. Stone, P.J. Hume, N.P. Greene, and V. Koronakis. 2020. Structure of CYRI-B (FAM49B), a key regulator of cellular actin assembly. Acta Crystallogr. D Struct. Biol. 76:1015–1024. 10.1107/S2059798320010906 33021503 PMC7543656

[bib35] Kavran, J.M., D.E. Klein, A. Lee, M. Falasca, S.J. Isakoff, E.Y. Skolnik, and M.A. Lemmon. 1998. Specificity and promiscuity in phosphoinositide binding by pleckstrin homology domains. J. Biol. Chem. 273:30497–30508. 10.1074/jbc.273.46.30497 9804818

[bib36] Lai, C.L., A. Srivastava, C. Pilling, A.R. Chase, J.J. Falke, and G.A. Voth. 2013. Molecular mechanism of membrane binding of the GRP1 PH domain. J. Mol. Biol. 425:3073–3090. 10.1016/j.jmb.2013.05.026 23747485 PMC4265004

[bib37] Leslie, E.J., J.C. Carlson, J.R. Shaffer, E. Feingold, G. Wehby, C.A. Laurie, D. Jain, C.C. Laurie, K.F. Doheny, T. McHenry, . 2016. A multi-ethnic genome-wide association study identifies novel loci for non-syndromic cleft lip with or without cleft palate on 2p24.2, 17q23 and 19q13. Hum. Mol. Genet. 25:2862–2872. 10.1093/hmg/ddw104 27033726 PMC5181632

[bib38] Makyio, H., M. Ohgi, T. Takei, S. Takahashi, H. Takatsu, Y. Katoh, A. Hanai, T. Ueda, Y. Kanaho, Y. Xie, . 2012. Structural basis for Arf6-MKLP1 complex formation on the Flemming body responsible for cytokinesis. EMBO J. 31:2590–2603. 10.1038/emboj.2012.89 22522702 PMC3365427

[bib39] McDonald, J.H. 2014. Handbook of Biological Statistics. Third edition. Sparky House Publishing, Baltimore, MD.

[bib40] Meinhardt, H. 1999. Orientation of chemotactic cells and growth cones: models and mechanisms. J. Cell Sci. 112:2867–2874. 10.1242/jcs.112.17.2867 10444381

[bib41] Mierke, C.T., B. Frey, M. Fellner, M. Herrmann, and B. Fabry. 2011. Integrin α5β1 facilitates cancer cell invasion through enhanced contractile forces. J. Cell Sci. 124:369–383. 10.1242/jcs.071985 21224397 PMC3021998

[bib42] Millius, A., S.N. Dandekar, A.R. Houk, and O.D. Weiner. 2009. Neutrophils establish rapid and robust WAVE complex polarity in an actin-dependent fashion. Curr. Biol. 19:253–259. 10.1016/j.cub.2008.12.044 19200726 PMC2705202

[bib43] Mooren, O.L., B.J. Galletta, and J.A. Cooper. 2012. Roles for actin assembly in endocytosis. Annu. Rev. Biochem. 81:661–686. 10.1146/annurev-biochem-060910-094416 22663081

[bib44] Moreno-Layseca, P., N.Z. Jäntti, R. Godbole, C. Sommer, G. Jacquemet, H. Al-Akhrass, P. Kronqvist, R.E. Kallionpää, L. Oliveira-Ferrer, P. Cervero, . 2020. Cargo-specific recruitment in clathrin and dynamin-independent endocytosis. BioRxiv. (Preprint posted October 5, 2020.) 10.1101/2020.10.05.323295

[bib45] Nam, J.M., Y. Onodera, M.J. Bissell, and C.C. Park. 2010. Breast cancer cells in three-dimensional culture display an enhanced radioresponse after coordinate targeting of integrin alpha5beta1 and fibronectin. Cancer Res. 70:5238–5248. 10.1158/0008-5472.CAN-09-2319 20516121 PMC2933183

[bib46] Paul, N.R., J.L. Allen, A. Chapman, M. Morlan-Mairal, E. Zindy, G. Jacquemet, L. Fernandez del Ama, N. Ferizovic, D.M. Green, J.D. Howe, . 2015. α5β1 integrin recycling promotes Arp2/3-independent cancer cell invasion via the formin FHOD3. J. Cell Biol. 210:1013–1031. 10.1083/jcb.201502040 26370503 PMC4576860

[bib47] Pietilä, M., P. Sahgal, E. Peuhu, N.Z. Jäntti, I. Paatero, E. Närvä, H. Al-Akhrass, J. Lilja, M. Georgiadou, O.M. Andersen, . 2019. SORLA regulates endosomal trafficking and oncogenic fitness of HER2. Nat. Commun. 10:2340. 10.1038/s41467-019-10275-0 31138794 PMC6538630

[bib48] Rainero, E., J.D. Howe, P.T. Caswell, N.B. Jamieson, K. Anderson, D.R. Critchley, L. Machesky, and J.C. Norman. 2015. Ligand-Occupied Integrin Internalization Links Nutrient Signaling to Invasive Migration. Cell Rep. 10:398–413. 10.1016/j.celrep.2014.12.037 25600874

[bib49] Rizzo, M.A., M.W. Davidson, and D.W. Piston. 2009. Fluorescent protein tracking and detection: fluorescent protein structure and color variants. Cold Spring Harb. Protoc. 2009:top63. 10.1101/pdb.top63 20150100

[bib67] Roy, A., A. Kucukural, and Y. Zhang. 2010. I-TASSER: a unified platform for automated protein structure and function prediction. Nat. Protoc. 5(4):725–738. 10.1038/nprot.2010.5 20360767 PMC2849174

[bib50] Schink, K.O., K.W. Tan, H. Spangenberg, D. Martorana, M. Sneeggen, C. Campsteijn, C. Raiborg, and H. Stenmark. 2017. The PtdIns3P-binding protein Phafin2 escorts macropinosomes through the cortical actin cytoskeleton. BioRxiv. (Preprint posted August 25, 2017.) 10.1101/180760

[bib51] Schlam, D., R.D. Bagshaw, S.A. Freeman, R.F. Collins, T. Pawson, G.D. Fairn, and S. Grinstein. 2015. Phosphoinositide 3-kinase enables phagocytosis of large particles by terminating actin assembly through Rac/Cdc42 GTPase-activating proteins. Nat. Commun. 6:8623. 10.1038/ncomms9623 26465210 PMC4634337

[bib52] Schliwa, M. 1982. Action of cytochalasin D on cytoskeletal networks. J. Cell Biol. 92:79–91. 10.1083/jcb.92.1.79 7199055 PMC2112008

[bib53] Shang, W., Y. Jiang, M. Boettcher, K. Ding, M. Mollenauer, Z. Liu, X. Wen, C. Liu, P. Hao, S. Zhao, . 2018. Genome-wide CRISPR screen identifies FAM49B as a key regulator of actin dynamics and T cell activation. Proc. Natl. Acad. Sci. USA. 115:E4051–E4060. 10.1073/pnas.1801340115 29632189 PMC5924929

[bib54] Shi, F., and J. Sottile. 2008. Caveolin-1-dependent beta1 integrin endocytosis is a critical regulator of fibronectin turnover. J. Cell Sci. 121:2360–2371. 10.1242/jcs.014977 18577581 PMC2587120

[bib55] Swanson, J.A., and C. Watts. 1995. Macropinocytosis. Trends Cell Biol. 5:424–428. 10.1016/S0962-8924(00)89101-1 14732047

[bib56] Timpson, P., E.J. McGhee, Z. Erami, M. Nobis, J.A. Quinn, M. Edward, and K.I. Anderson. 2011. Organotypic collagen I assay: a malleable platform to assess cell behaviour in a 3-dimensional context. J. Vis. Exp. (56):e3089. 10.3791/3089 22025017 PMC3227204

[bib57] Várnai, P., and T. Balla. 1998. Visualization of phosphoinositides that bind pleckstrin homology domains: calcium- and agonist-induced dynamic changes and relationship to myo-[3H]inositol-labeled phosphoinositide pools. J. Cell Biol. 143:501–510. 10.1083/jcb.143.2.501 9786958 PMC2132833

[bib58] Várnai, P., T. Bondeva, P. Tamás, B. Tóth, L. Buday, L. Hunyady, and T. Balla. 2005. Selective cellular effects of overexpressed pleckstrin-homology domains that recognize PtdIns(3,4,5)P3 suggest their interaction with protein binding partners. J. Cell Sci. 118:4879–4888. 10.1242/jcs.02606 16219693

[bib59] Veltman, D.M., T.D. Williams, G. Bloomfield, B.C. Chen, E. Betzig, R.H. Insall, and R.R. Kay. 2016. A plasma membrane template for macropinocytic cups. eLife. 5:e20085. 10.7554/eLife.20085 27960076 PMC5154761

[bib70] Weiner, O.D., W.A. Marganski, L.F. Wu, S.J. Altschuler, and M.W. Kirschner. 2007. An Actin-Based Wave Generator Organizes Cell Motility. PLoS Biol. 5(9):e221. 10.1371/journal.pbio.0050221 17696648 PMC1945041

[bib68] Yang, J., and Y. Zhang. 2015. Protein Structure and Function Prediction Using I‐TASSER. Curr. Protoc. Bioinformatics. 52(1). 10.1002/0471250953.bi0508s52 PMC4871818 26678386

[bib60] Yarmola, E.G., T. Somasundaram, T.A. Boring, I. Spector, and M.R. Bubb. 2000. Actin-latrunculin A structure and function. Differential modulation of actin-binding protein function by latrunculin A. J. Biol. Chem. 275:28120–28127. 10.1074/jbc.M004253200 10859320

[bib61] Yelland, T., A.H. Le, S. Nikolaou, R. Insall, L. Machesky, and S. Ismail. 2021. Structural Basis of CYRI-B Direct Competition with Scar/WAVE Complex for Rac1. Structure. 29:226–237.e4. 10.1016/j.str.2020.11.003 33217330 PMC7955166

[bib62] Yoshida, S., A.D. Hoppe, N. Araki, and J.A. Swanson. 2009. Sequential signaling in plasma-membrane domains during macropinosome formation in macrophages. J. Cell Sci. 122:3250–3261. 10.1242/jcs.053207 19690049 PMC2736863

[bib63] Yu, X., T. Zech, L. McDonald, E.G. Gonzalez, A. Li, I. Macpherson, J.P. Schwarz, H. Spence, K. Futó, P. Timpson, . 2012. N-WASP coordinates the delivery and F-actin-mediated capture of MT1-MMP at invasive pseudopods. J. Cell Biol. 199:527–544. 10.1083/jcb.201203025 23091069 PMC3483131

[bib64] Yuki, K.E., H. Marei, E. Fiskin, M.M. Eva, A.A. Gopal, J.A. Schwartzentruber, J. Majewski, M. Cellier, J.N. Mandl, S.M. Vidal, . 2019. CYRI/FAM49B negatively regulates RAC1-driven cytoskeletal remodelling and protects against bacterial infection. Nat. Microbiol. 4:1516–1531. 10.1038/s41564-019-0484-8 31285585

[bib65] Zech, T., S.D. Calaminus, P. Caswell, H.J. Spence, M. Carnell, R.H. Insall, J. Norman, and L.M. Machesky. 2011. The Arp2/3 activator WASH regulates α5β1-integrin-mediated invasive migration. J. Cell Sci. 124:3753–3759. 10.1242/jcs.080986 22114305 PMC3225265

[bib69] Zhang, Y. 2008. I-TASSER server for protein 3D structure prediction. BMC Bioinformatics. 9(1). 10.1186/1471-2105-9-40 PMC2245901 18215316

